# Genetic Landscape of Papillary Thyroid Carcinoma and Nuclear Architecture: An Overview Comparing Pediatric and Adult Populations

**DOI:** 10.3390/cancers12113146

**Published:** 2020-10-27

**Authors:** Aline Rangel-Pozzo, Luiza Sisdelli, Maria Isabel V. Cordioli, Fernanda Vaisman, Paola Caria, Sabine Mai, Janete M. Cerutti

**Affiliations:** 1 Cell Biology, Research Institute of Oncology and Hematology, University of Manitoba, CancerCare Manitoba, Winnipeg, MB R3E 0V9, Canada; aline.rangelpozzo@umanitoba.ca; 2 Genetic Bases of Thyroid Tumors Laboratory, Division of Genetics, Department of Morphology and Genetics, Universidade Federal de São Paulo/EPM, São Paulo, SP 04039-032, Brazil; l.sisdelli@unifesp.br (L.S.); isabel.cordioli@unifesp.br (M.I.V.C.); j.cerutti@unifesp.br (J.M.C.); 3 Instituto Nacional do Câncer, Rio de Janeiro, RJ 22451-000, Brazil; fvaisman@inca.gov.br; 4 Department of Biomedical Sciences, University of Cagliari, 09042 Cagliari, Italy

**Keywords:** Papillary thyroid carcinoma, BRAF^V600E^, pediatric, nuclear architecture, RET/PTC, AGK-BRAF, genomic instability

## Abstract

**Simple Summary:**

Papillary thyroid carcinoma (PTC) represents 80–90% of all differentiated thyroid carcinomas. PTC has a high rate of gene fusions and mutations, which can influence clinical and biological behavior in both children and adults. In this review, we focus on the comparison between pediatric and adult PTC, highlighting genetic alterations, telomere-related genomic instability and changes in nuclear organization as novel biomarkers for thyroid cancers.

**Abstract:**

Thyroid cancer is a rare malignancy in the pediatric population that is highly associated with disease aggressiveness and advanced disease stages when compared to adult population. The biological and molecular features underlying pediatric and adult thyroid cancer pathogenesis could be responsible for differences in the clinical presentation and prognosis. Despite this, the clinical assessment and treatments used in pediatric thyroid cancer are the same as those implemented for adults and specific personalized target treatments are not used in clinical practice. In this review, we focus on papillary thyroid carcinoma (PTC), which represents 80–90% of all differentiated thyroid carcinomas. PTC has a high rate of gene fusions and mutations, which can influence the histologic subtypes in both children and adults. This review also highlights telomere-related genomic instability and changes in nuclear organization as novel biomarkers for thyroid cancers.

## 1. Introduction

Thyroid carcinoma is the most common malignancy of the endocrine system in adult and pediatric populations. In adults, this type of cancer is increasing dramatically in both men and women, with an average annual percentage change of 5.4% and 6.5%, respectively. It is projected to take the place of colon cancer and become the fourth leading cancer diagnosis in both sexes (second for women) by 2030 [1,2]. Thyroid cancer presents with relatively stable mortality, but it has been increasing globally since the 1970s [3,4,5,6,7,8,9,10,11,12,13,14,15,16,17,18]. It is estimated that by the end of the year 2020, thyroid cancer will claim the lives of 2180 of the 52,890 new projected cases, corresponding to 0.4% of all cancer related deaths and 2.9% of new cancers throughout the world, respectively [19].

In the pediatric population (≤18 y.o. at diagnosis), thyroid cancer corresponds to 6% of all pediatric cancers (2012–2016 data) [20,21]. Even though there is no indication of ethnic or race susceptibility in pediatric thyroid cancer, there has been a prevalence related to increasing age range, i.e., ages 5–9, 10–14, and 15–19 showing a prevalence of 10,000, 80,000, and 310,000, respectively [19]. Considering gender, the prevalence is observed above age 10, and females are the most affected (more precisely between ages 13 and 19) [22,23,24]. Overall, among adolescents (ages 15–19), thyroid carcinoma is the eighth most diagnosed cancer [25,26].

Differentiated thyroid carcinoma (DTC) originates in the follicular cells of the thyroid and is the most common type (80–90%) of thyroid malignancy [27]. DTC is classified into follicular thyroid carcinoma (FTC) and papillary thyroid carcinoma (PTC). This classification relies on histological differences and the different metastatic dissemination routes between the two subtypes. FTC accounts for 10% of all DTC and is characterized by the presence of small follicles and the absence of ground-glass nuclei (characteristic of PTC). PTC encompasses the remaining 80–90% of all DTC and is characterized mainly by the presence of cells arranged into papillae, presenting clear or ground-glass nuclei. PTC is further subdivided based on histological variants, such as the classic (CVPTC), follicular (FVPTC), solid (SVPTC), and diffuse sclerosing (DSVPTC) variants. Among these variants, children under the age of 10 seem to be unaffected by the most common type, CVPTC, found in adults [26].

Oddly enough, regardless of studies suggesting that clinical presentation, pathophysiology, and long-term outcomes diverge between pediatric and adult populations, clinical assessment and treatment recommendations used in pediatric thyroid cancer are the same as those implemented for adults [21,28,29,30,31,32,33,34,35,36,37]. Looking closely, PTC differences in these populations could be explained by the distinct genetic alterations observed in the PTC of adults and children.

In this review, we will discuss aspects of the PTC histotype in adults and children, with a focus on differences in genetic alterations, telomere-related genomic instability, and nuclear architecture.

## 2. Epidemiology and Pathogenesis

**Figure 1:**
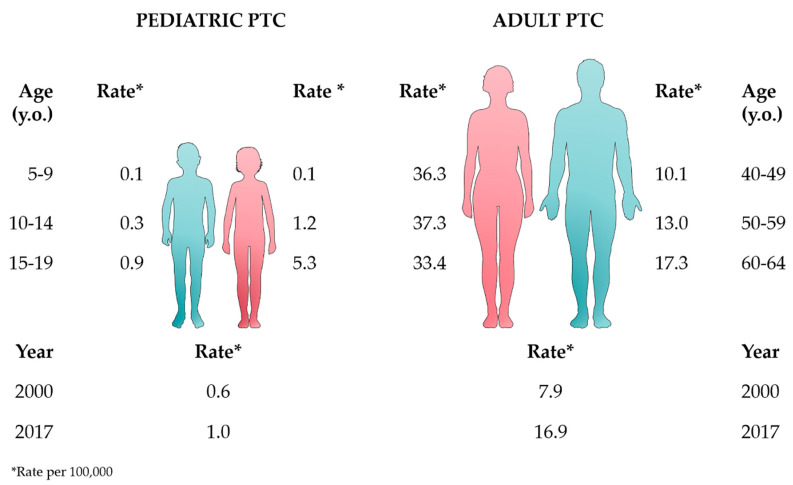
Epidemiologic data from the Surveillance, Epidemiology, and End Results (SEER) database (2000–2017) [19] comparing the rates of pediatric and adult papillary thyroid carcinoma (PTC) according to age, gender, and year. This figure was created using images from Servier Medical Art (http://smart.servier.com). Servier Medical Art by Servier is licensed under a Creative Commons Attribution 3.0 Unported License.

According to the Surveillance, Epidemiology, and End Results (SEER) database, the incidence of PTC in adults increased between 2000 and 2017, from 7.9 to 16.9 per 100,000, compared to 0.6 to 1.0 per 100,000 in the pediatric group (Figure 1, bottom lines) [19]. Remarkably, as represented in Figure 1, PTC in adults occurs more commonly in women at aged 50–59 (37.3 × 100,000) and to a lower rate (17.3 × 100,000) in men, for whom the peak of incidence occurs at ages 65–69. Looking at the pediatric population, this difference in gender starts just above age 10, i.e., 0.3 per 100,000 for boys and 1.2 per 100,000 for girls (ages 10–14), with increasing distinction above age 15, where the incidence increases to 0.9 per 100,000 for boys vs. 5.3 per 100,000 for girls (ages 15–19) (Figure 1) [19].

The reasons associated with this progressive trend are controversial. Several authors propose that the increase in cases is due to better diagnosis, since this tendency coincides with the increased use of high resolution imaging techniques [3,8,38,39,40]. Others suggest that the reason is multifactorial and is related to environmental and lifestyle factors. Diet, obesity, smoking, drinking, sex hormones, iodine deficiency, and a history of benign nodules in the family may contribute to the increased PTC incidence [41,42,43,44].

In the pediatric population, the only consolidated risk factor is the exposure to radiation in childhood, either environmental or as part of radiotherapy for a prior malignancy or treatment for another benign condition [45,46]. In fact, several studies have demonstrated a much greater sensitivity to radiation in children compared with adults. In the past 60 years, the incidence of pediatric cases peaked twice. The first peak occurred in the 1950s, due to the use of external irradiation of the head and neck to treat children with various benign non-thyroid disorders such as the enlargement of the thymus, tinea capitis, adenoids or neck lymph nodes, acne, eczema, otitis, and others [45,46,47]. The use of external radiation therapy on the neck essentially ended in the early 1960s, when a cause–effect relationship between radiation exposure and PTC was established [45,46,47]. However, radiation is still used in clinical practice to treat different types of cancers. Radiation-induced malignancies, such as thyroid cancer, are late complications of radiotherapy treatment, with increased frequency among survivors of both pediatric and adult cancers [48].

Although there was a sharp increase in the incidence of childhood thyroid cancer in the Minsk and Kiev centers 4–5 years after the explosion of the Chernobyl Nuclear Power Plant reactors in 1986, the second peak of incidence occurred just 10 years after the accident in some Eastern European countries. The high-risk group comprised children under the age of four at the time of exposure. Consequently, in this second peak, the majority of clinically evident tumors were present in children ~10–14 years old [22,24,26]. Regarding the Fukushima Daiichi nuclear disaster (March 2011), it is still unclear whether the radiation released after the nuclear accident could be considered the cause of a “third peak” of thyroid cancer incidence in the pediatric group, or if a potential peak is just an artefactual result of the intense screening of this population. The adverse effects of the Fukushima accident might have been partially mitigated by the measures taken, i.e., evacuation from most of the contaminated areas and the recommendation of a low iodine alimentary intake and food restrictions, which could have reduced the uptake of iodine-131. With an average radiation dose of < 1 mSv for the majority of Fukushima residents and a maximum of 30 mSv in few cases from evacuated sites near to the Fukushima Nuclear Power Plant, the first round of thyroid ultrasound screening, performed in all affected children under age 18, showed no clear evidence of a thyroid cancer increase due to radiation exposure [49]. Other studies have found a significant dose–response relationship between the rate of thyroid cancer detection and the external effective dose-rate in both the first and second rounds of the thyroid ultrasound screening [50,51]. The third and the fourth rounds of examinations are still in progress and further data may bring more light into this issue. Interestingly, as discussed in the next section, the pathological findings observed in the Fukushima PTC cases are similar to the pediatric cases found in non-exposed areas and to the mutational profile reported in adult PTC [52,53].

## 3. Clinical Features, Prognosis, and Treatment

The differences in clinical presentation and outcomes between pediatric and adult PTC are significant [54,55,56]. Compared to those of adults, pediatric thyroid cancers usually present with more advanced disease. Though the recurrence rates are higher than in adults, pediatric PTC has a better long-term outcome, with minimal or no mortality in most cases [54,57,58]. Pediatric PTC typically manifests as a palpable thyroid nodule/tumor, with or without cervical lymphadenopathy [59]. Although rare in children and adolescents, the presence of nodules in pediatric patients is clinically important. Thyroid nodules are associated with increased malignancy compared to adults (26% vs. 5%) [60,61]. Additionally, the mean tumor size is typically larger in pediatric patients. Hay et al. (2018) studied 190 children and 4242 adults consecutively treated during 1936–2015. They described a mean tumor size of 2.56 cm (median = 2.15 cm) in children vs. 1.94 cm (median = 1.5 cm) in adult patients [56]. Papillary thyroid microcarcinoma (≤1 cm) accounts for ~40% of tumors in adults [62] and represents < 10% of pediatric PTC [63]. This difference is probably due to the common practice of thyroid cancer screening in adults and the early detection of smaller tumors [64].

Furthermore, when compared to adults, childhood thyroid carcinomas are more frequently locally invasive. The metastatic involvement of regional lymph nodes at diagnosis was reported in ~50–75% of pediatric cases (Table 1) [55,56,65,66], compared to ~20–40% in adult PTC [56,66]. With respect to distant metastasis, data available from the literature also demonstrate a high frequency in pediatric vs. adult PTC patients [56]. The lungs are the most common site of distant metastases in all age groups, occurring in ~5–16% of pediatric PTC (Table 1) and in 2–4% of adults [54,55,56]. Liu et al. (2019) investigated the occurrence of factors influencing distant metastasis in pediatric thyroid cancer and identified the age at diagnosis as an important factor, with distant metastasis occurring in 1.73% of patients aged 15 and above, and in 6.73% of patients under the age of 15 [67].

Despite the higher rate of disease recurrence when compared to adults, overall survival is higher in pediatric PTC [29,77]. Mazzaferri et al. (2001) [78], in a series of 16.6 years’ follow-up, found a disease recurrence rate of ~40% in patients under the age of 20 and ~20% in patients above the age of 20. Additionally, Demidchik et al. (2006) [79], with a cohort of 741 patients, found a survival rate of 99.3% at age 5 and 98.5% at age 10. Lazar et al. (2009) [80] demonstrated that patients under the age of 10, mainly pre-pubertal patients, presented a worse prognosis than older ones or those in more advanced puberty stages. It seems that large tumors (>2 cm), extra-thyroidal extension, and younger age are factors associated with worse prognosis. However, the ideal cut-off for age and pubertal status awaits future investigation. The same is true for gender, which two studies showed to be an important prognostic factor [70,72], whereas another study showed no significance [67].

PTC treatment is based on the combination of three therapeutic modalities: surgery, radioiodine therapy, and hormone replacement with levothyroxine. Surgery can range from lobectomy to total thyroidectomy, accompanied by cervical lymphadenectomy. The extent of thyroid surgery for adult PTC patients has shifted in a more conservative direction in most recent guidelines [30]. Since then, lobectomy has been an acceptable surgical treatment for low-risk tumors without extrathyroidal extension or clinical lymph node metastases. However, the American Thyroid Association (ATA) management guidelines for children with PTC recommend total thyroidectomy for the majority of children [21,30]. The rationale for this approach is based on an increased incidence of bilateral and multi-focal disease in pediatric patients. It consists of the dissection of the central cervical compartment, with the removal of lymph nodes and adjacent tissues suspected to present metastasis. Modified lateral cervical dissection is indicated in cases of metastasis to lateral lymph nodes. The main surgical complications include persistent hypoparathyroidism and injury to the recurrent laryngeal nerve, which can cause hoarseness to complete closure of the vocal cords, requiring a definitive tracheostomy [81,82]. Fridman et al. (2019) [83] have reported a number of complications of thyroid surgery in childhood PTC. However, they concluded that prophylactic neck dissections should be recommended in children and adolescents due to the high rates of node metastases. On the other hand, to avoid surgical morbidity, Francis et al. (2015) [21] proposed that surgery for pediatric patients should take into account the risk stratification variables, in which patients are divided into a low, intermediate, and high risk of recurrence.

After total or almost total thyroidectomy, the volume of the remaining gland must be <2 mL at cervical ultrasound, performed up to 1 month after surgery [77,84]. Interestingly, even after total thyroid removal, with no thyroid detected by ultrasound, radioiodine (RAI) uptake in the thyroid bed occurs [85]. This phenomenon is usually attributed to remaining thyroid cells. However, since multifocality and metastasis are more common in the pediatric age group, the possibility that such foci still have malignant cells cannot be ruled out. Despite this, most societies recommend the ablation of reminiscent tissue in the majority of pediatric patients [21]. The pediatric recommendations regarding indications for RAI are still controversial. The National Comprehensive Cancer Network for adults suggests clinical features including tumor size >2–4 cm, gross extrathyroidal extension, and extensive regional nodal involvement as indicators for adjuvant RAI [86]. The guidelines for children recommend an individualized approach using post-operative thyroid-stimulating hormone (TSH)-stimulated thyroglobulin levels to determine who should receive adjuvant RAI [21]. There is no consensus in the calculation of the appropriate dose of iodine-131 (^131^I) for pediatric patients, since both body weight and body surface area methods are used. Whole body ^131^I dosimetry can also be used in patients with extensive metastases [87]. The success rate of ablation is significantly lower in patients who have undergone less extensive surgery, whether they are children or adults [22,78,84].

Successful ablation is usually defined as the absence of uptake or uptake of less than 0.1–1%, as detected by means of a total body scintigraphy performed 6–12 months after the procedure [85,88,89], accompanied by markedly decreased or undetectable serum thyroglobulin, and suboptimal TSH stimulus, all happening at the same time [77,78,88]. In most cases, one dose of radiodine therapy is able to achieve these goals [85], if not, the procedure may be repeated no earlier than 12 months after the first attempt [88,89]. The ablation should also be followed by a total body scintigraphy (post-therapeutic whole-body scan), performed ~5–7 days after the administration of the radioiodine, in order to detect or confirm the presence of functional metastases.

Lastly, thyroid hormone replacement, the third treatment modality, involves the oral use of levothyroxine. This modality is called suppressive therapy with thyroid hormone when a supraphysiological dose is used in order to keep serum TSH levels below the lower reference limit, reducing the risk of TSH-induced tumor growth or proliferation [90]. In children and adolescents, there are several studies guaranteeing the effectiveness and safety of this type of replacement, as long as it is carefully controlled, particularly regarding the patient’s final height [66,77,91]. The actual recommendation is to keep TSH suppressed as needed [21]. Possible side effects of long-term suppressive therapy, documented in adults, include osteoporosis [82] and cardiovascular diseases, especially left ventricular hypertrophy [92,93]. Regarding fertility, some studies suggest that radioiodine may affect testicular and ovarian function, at least temporarily [94,95,96].

## 4. Molecular Features

Different molecular markers of diagnosis, prognosis, treatment, and follow-up have been identified in PTC [97,98]. In adults, the most common genetic alterations are *BRAF*^V600E^ and *RAS* point mutations and *RET/PTC* fusions (Table 2) [99,100]. Moreover, *hTERT* promoter mutations were observed in adult PTC and are associated with a more aggressive phenotype [101].

In 2014, The Cancer Genome Atlas (TCGA) performed an analysis of nearly 500 PTCs from adult patients [100]. It not only confirmed the presence of *BRAF*^V600E^ (59.7%) and *RAS* (13%) mutations and *RET* (6.3%) fusion in most PTCs, but also revealed new driver genes such as *EIF1AX* (1.5%), *PPM1D* (1.2%), and *CHEK2* (1.2%) [100]. Based on gene expression profiles, PTC was further divided in two highly distinct classes that display distinctive differentiation and signaling properties: *BRAF*^V600E^-like and *RAS*-like PTCs. *BRAF*^V600E^-like tumors are predominantly characterized by *BRAF*^V600E^ mutations and *BRAF, RET,* and *NTRK1/3* fusions and show preeminent activation of the mitogen activated protein kinase (MAPK) signaling pathway. *RAS*-like tumors are predominantly characterized by *H/N/K-RAS*, *EIF1AX,* and *BRAF*^K601E^ point mutations and *PPARG* fusions and are activated by both the MAPK and PI3K/AKT signaling pathways. Even though the two groups are highly correlated, they were derived independently and have no genes in common [100].

On the other hand, in pediatric PTC, there is higher incidence of *RET/PTC*, *ETV6-NTRK3* and *BRAF* fusions (*AGK-BRAF* and *AKAP9-BRAF*), mainly in patients under the age of 10. *BRAF^V600E^* mutation is less common, and *RAS* and *hTERT* promoter mutations are rarely found in the pediatric population (Table 2) [117,120,121,124,161]. Indeed, studies show that nearly 50% of pediatric tumors harbor some type of rearrangement, regardless of the radiation exposure [120,134]. As pediatric PTC exhibits a distinct genetic background, it is not usually classified into *BRAF^V600E^*-like and *RAS*-like nodules (Table 2).

It is important to note that most studies performed in both pediatric and adult PTC reported in this review investigated the molecular features of thyroid cancer before the nomenclature revision of an encapsulated follicular variant of PTC subset in 2016 as non-invasive follicular thyroid neoplasm with papillary-like nuclear features (NIFTP) [162]. Therefore, this new entity was considered as a PTC. The exclusion of these tumors from the molecular studies of thyroid cancer would certainly change the prevalence of genetic events described in thyroid cancer, both adult and pediatric, since the mutational profile of NIFTP is still unidentified but resembles that of follicular thyroid adenoma (FTA), with *RAS* mutations and *PAX8-PPARγ* fusion [152,163].

### 4.1. BRAF Alterations

The B-Raf (*BRAF*) gene is a member of the Raf family of serine/threonine protein kinases located in 7q34 [103]. In PTC from the adult population, the most prevalent mutation is found within exon 15 of the *BRAF* gene. The thymine to adenine transversion at nucleotide position 1799 (T1799A), which results in a valine to glutamate substitution at residue 600 (V600E), occurs in about 27–83% of PTC cases (Table 2) [100,102,103,104,105,106,107]. The BRAF ^K601E^ point mutation, which display lower oncogenic activity than *BRAF*^V600E^ in vitro, is more frequently associated with FVPTC [164]. In the adult population, fusions involving the *BRAF* gene with different partners were found in nearly 2.3% of PTC samples from TCGA study group, being *AGK-BRAF* found in 1 (0.2%) PTC sample [100].

In the pediatric population, *BRAF*^V600E^ is rarely found in radiation-exposed PTC samples (Table 2). However, in the post-Fukushima PTC samples, *BRAF*^V600E^ was detected in ~70% of the tumors [52,126]. In sporadic pediatric PTC, *BRAF*^V600E^ mutations have been found at different frequencies, varying from 0% to 63% (Table 2) [26,109,110,111,112,113,114,116,117,118,119,120,121,122,123]. The difference in allele frequencies might reflect the age of patients; geographical, racial, ethnic differences, environmental factors; and methodological approaches [114,116,160]. In fact, different studies have demonstrated that the number of *BRAF*^V600E^-positive tumors increases with age [116,160].

A-kinase anchoring protein 9 (*AKAP9)-BRAF* fusion, which is a result of the paracentric inversion inv(7)(q21q34), was first identified in post-Chernobyl pediatric PTC (Table 2) [117,124,125] but has been also observed in adult PTC (Table 2) [100,124].

Another important *BRAF* fusion is acylglycerol kinase (*AGK*)-*BRAF* fusion, which is also a result of a paracentric inversion inv (7)(q34), juxtaposing exons 1–2 of *AGK* to exons 8–18 of *BRAF*. This rearrangement was first identified in a radiation-exposed patient from Ukraine, and later was observed in sporadic pediatric (19%) and adult PTC cases (0–0.2%) (Table 2) [100,115,117,119,121,127,128,129,130,131,161]. Remarkably, *AGK-BRAF* fusion in sporadic pediatric patients can differ geographically. We have found *AGK-BRAF* in 19% of the sporadic PTC Brazilian patients [121]; however, the fusion was not observed in any pediatric PTC cases from the US or the Czech Republic (Table 2) [129,130,131,134]. In radiation-exposed PTC Ukrainian pediatric cases, *AGK-BRAF* fusion was described in 2% of PTC cases [117,125].

These *BRAF* alterations (mutations and fusions) lead to a constitutive activation of the BRAF kinase and MAPK pathways, which are predominantly implicated in the pathogenesis of PTC [99]. Neither *BRAF*^V600E^ nor *BRAF* fusions have been described in follicular thyroid carcinomas or benign nodules, reinforcing its association with the PTC subtype.

Another study reported a direct association between *BRAF*^V600E^ and disease aggressiveness in adult PTC alone [165], but this association is unclear in pediatric PTC. Moreover, it has been shown that *BRAF*^V600E^ mutation is associated with larger tumor sizes (>2 cm) in both pediatric and adult PTC [121,166,167], but findings showing an intratumor genetic heterogeneity involving *BRAF* mutation show contradictory results as to its prognostic value [101,165]. However, *BRAF*^V600E^ has not been described in other follicular carcinomas or benign nodules, which suggests that this mutation is strongly associated with PTC [104,153]. Both *AKAP9-BRAF* and *AGK-BRAF* fusion are capable of transforming NIH3T3 cells (fibroblast cell line) and continuously activate the MAPK pathway [117,124]. Although there is a relationship between *AKAP9-BRAF* and pediatric PTC clinical-pathological features, *AGK-BRAF* has been associated with lung metastasis [121,124].

Novel *BRAF* fusions (*OPTN-BRAF, CUL1-BRAF*) were described in two sporadic pediatric PTC cases from the Czech Republic [131]. Interestingly, several novel fusions involving the *BRAF* gene (*SND1-BRAF, MACF-BRAF, MBP-BRAF, POR-BRAF, ZBTB8A-BRAF*) have been described in Ukrainian-American patients that were under the age of 18 at the time of the Chernobyl accident [125] (Table 2).

### 4.2. RET/PTC Rearrangements

The rearranged during transfection (*RET*) gene is located in the long arm of chromosome 10 (10q11.2) and encodes for the tyrosine kinase receptor [168]. *RET* is normally expressed in the adrenal medulla and cerebellum among adult human tissues and in urogenital and neural crest cells during development, whereas it is absent in normal thyroid epithelium [169,170,171]. *RET* rearrangements lead to the activation of *RET* gene, once the rearrangement juxtaposes the kinase domain under the control of the transcriptional promoter of the fusion partners, expressed in normal follicular thyroid cells, leading to its constitutional activation. To date, over 20 *RET* fusions have been described, either as a result of 3’ kinase fusion (juxtaposition of the N-terminal partner to the C-terminal portion of the RTK) or 5’ kinase fusion (juxtaposition of the N-terminal portion of the RTK to the C-terminal of a fusion partner) [133].

The most common *RET* rearrangements are *RET/PTC1, RET/PTC2,* and *RET/PTC3*, where *RET* proto-oncogene fuses to the genes *H4* (10q21), *PRKAR1A* (17q23), and *NOCA4* (10q11.2), respectively [172]. In the general population the incidence of *RET/PTC* is ~10–25%, but it varies considerably among populations and could account for 50–70% of genetic alterations found in PTC samples (Table 2) [132]. This variability is likely due to different methods of detection; genetic heterogeneity of the tumor; or ethnical, racial, and geographical variations [100,173,174].

Childhood accidental or therapeutic exposures to ionizing radiation have been associated with *RET/PTC* rearrangements [172,175]. In fact, *RET/PTC* rearrangements are observed in 33–76% (average of 58%) of the radiation-exposed PTC cases and in about 22–65% of sporadic pediatric PTC cases (Table 2) [26,87]. *RET/PTC1* and *RET/PTC3* are the most common rearrangements found in the pediatric population. Interestingly, in adults, *RET* rearrangements usually have a favorable prognosis and a good response to radioactive iodine (RAI) therapy. However, in pediatric patients, some studies reported *RET* fusions with extrathyroidal extension, lymph node and lung metastasis, more aggressive variants, and poor prognosis [134,174,176,177,178]. It is still not clear what influences this prognostic difference in children and adults. Despite being considered a diagnostic molecular biomarker for PTC, *RET* fusions have also been described in benign thyroid lesions [179,180], in which case the rearrangements can be used as initial markers of early tumorigenesis.

Recently, novel *RET* fusions were reported in sporadic and radiation-exposed pediatric PTC. The *AFAP1L2-RET, PPFIBP-RET, KIAA1217-RET,* and *ΔRFP-RET* fusions were reported in nearly 3% of pediatric PTC cases from Fukushima [126]. Three novel *RET* fusions (*TPR-RET, IKBKG-RET, BBIP1-RET*) were described in nearly 3% of sporadic pediatric PTC cases from the Czech Republic [131] (Table 2).

### 4.3. ETV6-NTRK3 Rearrangement

*ETV6-NTRK3* gene fusion is a consequence of the t(12;15)(p13;q25) translocation and mainly exhibits two isoforms: *ETV6-NTRK3_1* (Cosmic ID: COSF1535) and *ETV6-NTRK3_2* (Cosmic ID: COSF1537), which corresponds to the fusion of exon 4 or exon 5 of *ETV6* with exon 14 of *NTRK3*, respectively. This fusion forms a chimeric oncoprotein that activates both the MAPK and PI3K/AKT pathways [117]. The *ETV6-NTRK3* fusion frequency in PTC is 1.2% according to TCGA (The Cancer Genome Atlas) analysis, but other studies reported this fusion in ~5% of adult PTC cases [127,145]. In pediatric PTCs, the fusion is common in the form of *RET/PTC* [115,117,120,131]. *ETV6-NTRK3* was first described in radiation-induced tumors and sporadic PTC cases from Ukraine [117] and later was found in radiation-exposed (14.5% of post-Chernobyl PTC patients aged 14–32) and sporadic cases (2% of patients aged 15–97) PTC cases [146] (Table 2) [120,131]. The prognosis significance and the possible association with age remain unclear in PTC.

*NTRK3* fusions are not limited to the aforementioned examples. Different *NTRK3* fusions (*RBPMS-NTRK3, EML4-NTRK3, SQTSM-NTRK3*, and *TPM3-NTRK3*) have been identified in sporadic pediatric PTC [115,131] and in radiation-exposed PTC (*SQTSM-NTRK3*) [139], but their significance will be revealed as more research efforts accumulate.

### 4.4. STRN-ALK Rearrangement

*STRN-ALK* rearrangement is a result of a complex rearrangement involving the short arm of chromosome 2, juxtaposing exon 3 of *STRN* to exon 20 of *ALK*. This fusion leads to constitutive activation of ALK kinase via dimerization mediated by the coiled-coil domain of the *STRN* gene, resulting in thyroid-stimulating hormone-independent proliferation of thyroid cells [148]. In addition, *STRN-ALK* expression was shown to be able to transform cells in vitro and induce tumor formation in mice [148]. Though rarely found in adult PTC (0.4–3% of cases) (Table 2) [100,127,148], this rearrangement was present in the advanced stage of the disease and dedifferentiated tumors, but with no clear prognostic significance. In pediatric PTC, *STRN-ALK* fusion is reported in 1.4–7% of radiation-exposed and 6.5% of sporadic cases (Table 2) [125,126,139,148].

### 4.5. PAX8-PPARγ Rearrangement

*PAX8-PPARγ* rearrangement results from the t(2;3)(q13;p25) translocation, which fuses exon 10 of the *PAX8* gene to exon 1 of *PPARγ*, leading to the constitutive activation of the PI3K/AKT pathway [181]. *PAX8-PPARγ* rearrangement is common in the adult PTC population but is rarely reported in pediatric PTC (0–9% of the sporadic cases and 4% of the radiation-exposed ones) (Table 2) [113,117,119,122,129,182]. This rearrangement is also observed in benign tumors, mainly FTA [183,184,185], with no clear role in prognosis.

### 4.6. RAS Mutations

RAS is a family of GTP-binding proteins that are key regulators of the MAPK and PI3K-AKT signaling pathways. Mutations in the GTP domain (codon 12–13) or GTPase (codon 61) produce a change in the amino acid sequence, resulting in its constitutive activation. The three genes of the family are *NRAS* (1p13.2), *HRAS* (11p15.5), and *KRAS* (12p12.1) [186]. In adult thyroid cancer, NRAS codon 61 (NRAS Q61K) and HRAS codon 61 (HRAS Q61R) mutations are the most frequent. They are observed in both benign and malignant thyroid nodules, including 10–20% of FVPTC cases (Table 2) [153]. On the other hand, *RAS* mutations are very rare in pediatric PTC and are observed in less than 5% of the sporadic tumors (Table 2) [111,113,119,120,134,187]. The prognostic significance of *RAS* mutations is also not clear, although some authors showed an association between *RAS* mutations and distant metastases in adult PTC [188].

## 5. Telomere-Related Genomic Instability and Nuclear Architecture

Telomeres, tandem repeats of the sequence (TTAGGG)n, ensure that the ends of chromosomes are not recognized as sites of DNA damage and are processed by DNA repair pathways [189,190]. Telomere function in humans depends on a cap of tightly bound proteins to repress DNA damage signaling, which includes the t-loop and the association of telomere-associated proteins, i.e., the shelterin complex—TRF1 and TRF2, POT1, TIN2, RAP1, and TPP1 [191]. Due to the inefficiency of the DNA replication machinery to replicate the chromosome ends, known as the end replication problem, telomeres progressively shorten after each cell division [192,193]. During replication, DNA synthesis of the discontinued strand at the replication fork occurs with a mechanism that produces short DNA fragments. However, this process meets a problem when the replication fork reaches the end of a linear chromosome/DNA. The final RNA primer synthesized on the discontinued-strand template cannot be replaced and telomere sequences are lost from the ends of all chromosomes each time a cell divides [192,193].

It is noteworthy that telomere shortening is an important tumor suppressor mechanism, as it leads to replicative cellular senescence and cycle arrest in normal cells, thus preventing genome instability. However, cancer cells can elongate their telomeres and regain telomere stability by activating one of two known telomere maintenance mechanisms (TMMs)—telomerase, which is activated in 85–90% of cancers; or the alternative lengthening of telomeres (ALT) mechanism (10–15% of cancers), which is often present in cancer cells that do not express telomerase [194].

However, some studies have indicated the coexistence of both ALT and telomerase activation, as well switching between TMMs in some tumor cells [195]. The co-existence of both TMM or telomerase/ALT switching has been observed in Wilms tumors [196], glioblastomas [197], gastric carcinomas [198], osteosarcomas [199], adrenocortical carcinomas [200], mesotheliomas [201], breast [202], and bladder cancers [203]. Bojovic et al. (2015) [204] demonstrated that ALT and telomerase activity coexist within the same cells, with possible competition between these two TMMs for telomere elongation. Telomerase activation and ALT switching in cancer was first described when tumor cells were treated with telomerase-targeted cancer drugs [195]. Those tumor cells are able to escape from cell death by switching from telomerase telomere extension to ALT. To date, the mechanisms underlying this switch between the two TMMs to maintain telomere length is not clear.

Given the key role of telomerase reverse transcriptase (*TERT*) in cancer, it is essential to understand the mechanism underlying telomerase activation and *TERT* expression. TERT activation can be promoted by translocations or amplification of the *TERT* promoter region, rather than by mutations alone or simply by de-repression of the *TERT* gene. During rearrangements, strong enhancers often juxtapose to the *TERT* coding sequence [205]. This event induces telomerase expression much more efficiently than *TERT* promoter mutations or amplifications. However, most of the studies on PTC focus on the identification of *TERT* promoter mutations. Indeed, overall, only 3% of all TERT-expressing tumor samples (adult and pediatric) present *TERT* amplification or translocations [206,207].

In adult PTC, mutations in the *TERT* promoter are more evident after malignant transformation, where 33% of the PTCs involved in distant metastasis display mutations in the TERT promoter [208]. The C228T and C250T mutations are the ones most commonly associated with aggressiveness, including advanced stage, larger tumor size, extrathyroidal invasion, metastasis, and disease recurrence [208]. Interestingly, C228T and C250T TERT promoter mutations are more prevalent in PTCs harboring *BRAF*^V600E^ mutation. This co-existence of *BRAF* and *TERT* is strongly associated with shorter progression free survival [108].

In pediatric PTC, fewer studies have reported *TERT* mutations or their association with prognosis. Geng et al. (2019) [209], with a cohort of 48 pediatric PTC patients, found a significant correlation between C228T mutation and disease aggressiveness. One important aspect of the study by Geng et al. (2019) [209] is the claim that TERT C250T mutation was not detected in the pediatric cohort. Even in adults, TERT C228T mutations are more prevalent than C250T TERT mutations. It is still not clear if the distribution of TERT promoter C250T mutations in pediatric PTC is rare or if due to the small sample sizes of the studies, researchers have underestimated the prevalence of C250T mutations in the pediatric PTC population.

However, even though *TERT* promoter mutation drives telomerase expression, it does not always prevent telomere shortening in PTC. This gave rise to the hypothesis that re-activated telomerase expression could only allow genetically unstable clones to maintain their telomeres barely above a critically short length, resulting in the prevention of cellular senescence and apoptosis [210]. Telomere shortening, genomic instability, and TERT activation are associated with features of PTC and are the most frequent alterations observed in aggressive stages [211,212]. Therefore, telomere studies could provide additional information to predict metastasis and aggressive behavior of PTC tumors having poor biological characterization and very limited therapeutic options.

With advances in 3D imaging analysis, it is now possible to analyze, on a single cell level, telomere length, telomere numbers, their spatial organization, and cell cycle dependency within interphase nuclei, rather than using the classical metaphase chromosomes, polymerase chain reactions, or DNA blotting techniques, which simply determine telomere length. Caria et al. (2019), used a 3D imaging approach to reveal specific 3D telomeric signatures of PTC-derived cell lines. This was the first study using 3D telomere quantitative fluorescence in situ hybridization and quantitative 3D imaging in PTC cells. The authors demonstrated that thyroid cell lines BCPAP, K1, and TPC1 (all with C228T mutation) have more telomere signals, more telomere aggregates, and less average intensity (proportional to telomere length) than the control cell lines Nthy-ori 3–1 [213]. Telomere aggregates are fused telomeric signals or telomeres in close illegitimate proximity that are capable of engaging in recombination events. Short and unprotected telomeres are recognized as broken DNA ends and are eventually joined by the DNA repair proteins, which may likely generate deletions, duplication, non-reciprocal translocation, and most of the overall genetic changes observed during tumor progression [211,212]. However, 3D nuclear organization in thyroid tumor sections from adult and pediatric patients remain underexplored.

Another feature of 3D fluorescent imaging is that it enables the ability to analyze the location of chromosomes in the 3D nuclear space. In normal cells, higher-order chromatin organization is necessary for proper genome function and regulation [181]. However, how the levels of organization are formed and the fundamental principles that guide interphase chromatin folding and unfolding are poorly described [181,182]. A general model for nuclear architecture can be illustrated as follows: first, chromosomes are arranged in a nonrandom organization in the nuclei of normal cells; second, chromosomes are placed into distinct territories and positions in the nuclear space; third, individual chromosomes can be folded together into open and active compartments (in the center of the nucleus) or closed and silent compartments (in the nuclear periphery) to control gene expression; and fourth, despite being in distinct neighborhoods, chromosomes can interact with other chromosomes that have been placed into a different territory [182]. In cancer cells, it seems that nuclear organization of chromosomes, and consequently gene position, can be reordered to modify gene expression [214]. The reallocation of chromosomes in different territories can enrich the oncogenic process, since chromosomes and genes commonly involved in cancer-associated translocations are thus able share the same nuclear localization where transcriptional and recombination factors are available [215,216,217]. These movements could also inactivate tumor suppressor genes or activate oncogenes simply by moving them to open/active or closed/silent nuclear compartments [215,216,217].

In PTC, the high rates of gene rearrangements are usually attributed to environmental radiation, although some genetic fusions are also found in sporadic cases. The spatial proximity of genes and chromosomes could explain the high rate of recurrence of rearrangement or inversions in pediatric PTC. Nikiforova et al. (2000) [218] visualized interphase distances between *RET* and *H4* genes in normal thyroid cells. They were found to be colocalized in at least one chromosome in 35% of adult thyroid cells. To classify them as colocalized, the authors reviewed 30 optical sections of each nucleus and signals were considered juxtaposed if they were touching each other or overlapping in at least one optical section. Gandhi et al. (2005) [219] also visualized colocalized *RET* to either *H4* or *NCOA4* in 25% and 34% of normal thyroid cells, respectively. This colocalization was measured using sensitized emission Förster resonance energy transfer (FRET) microscopy [220]. This technique is based on the energy transfer from a donor fluorophore to an acceptor. For this to occur, donor and acceptor molecules need to be within a distance of less than approximately 10 nm. *HRAS* and *RET/PTC* rearrangements are also known to affect chromatin structure but the mechanisms behind this process are still unknown. Changes in proteins involved in chromatin architecture, such as histones modifications, DNA methylation or chromatin remodeling, could also be associated with changes in gene-expression patterns observed in thyroid cancer cells [221].

**Figure 2:**
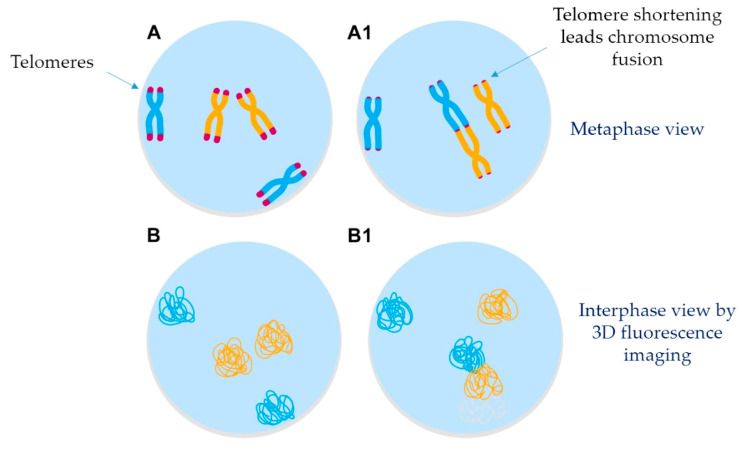
Model of chromosome reorganization in PTC. Changes in chromosome territories can reprogram gene expression. Some chromosomes localize toward the nuclear periphery, often touching the nuclear membrane, whereas others are located towards the center of the nucleus. In PTC, changes in chromosome territories can decrease the distance between genes, leading to a high rate of recurrence of specific chromosome rearrangements or inversions in PTC.

To conclude, the new nuclear organization could be used as a biomarker for thyroid cancers. If gene proximity and nuclear organization in chromosome territories are truly important for recombination and gene expression, it is expected that other rearrangements found in pediatric PTC, such as *RET*, *BRAF, NTRK*, and *ALK* fusion, are favored by this proximity or chromosome reorganization in thyroid cancer (Figure 2). Indeed, telomere shortening and uncapped chromosome ends in PTC could be responsible for randomly joined chromosomes that are in close spatial proximity. It is clear that 3D nuclear organization in thyroid cancer remains underexplored; therefore, more studies investigating the spatial nuclear signature that can be translated into biomarkers for the development and progression of thyroid cancer are critical. New therapeutic approaches could also emerge to revert malignancy-associated nuclear changes, with the potential to treat cancers with the involvement of multiple signaling pathways, such as aggressive thyroid cancer. A good example is nuclear structure promyelocytic leukemia (PML) bodies. The cancer drugs ATRA or As2O3 are able to promote the reformation of PML bodies in leukemia patients, leading to cell differentiation [221]. PML and PML bodies are not only affected in myeloid leukemia. PML overexpression (and cytoplasmic de-localization) has also been observed in PTC.

The nuclear architecture of cancer cells can also be analyzed in detail using 3D structured illumination microscopy (3D-SIM). The use of 3D-SIM is able to overcome the limits of conventional wide field fluorescence microscopy and reveal cellular structures that cannot be visualized directly by conventional microscopy [222]. Briefly, 3D-SIM uses illumination patterns to excite the sample and the reconstruction software doubles the resolution in all three dimensions [223,224,225]. The application of 3D-SIM has been geared to the study of biological structures, most importantly to analyze the chromatin present in cancer cells. Many authors [226,227,228,229,230,231] have used this technology to examine the cancer cell genome, where the presence of DNA structure, along with DNA-poor-spaces (spaces without DNA structure) were quantified. The biological significance of these poor spaces still needs further investigation, but they have been correlated with disease stage and tumor aggressiveness [226,227,228,229,230,231].

## 6. Conclusions and Future Perspectives

In this review, we summarized the genetic landscape of adult and pediatric PTC, discussing post-Chernobyl and post-Fukushima pediatric cases.

Although the genetic profile clearly depends on geographical localization, the central role of mutations of genes leading to constitutive activations of the mitogen-activated protein kinase (MAPK) pathway in the pathogenesis of PTC has to be acknowledged.

Although the genetic mechanism and the genes involved diverges considerably among populations, a strong genotype–phenotype correlation has been observed. *BRAF*^V600E^ confers a growth advantage in adults, but does not seem to confer the same biological capabilities in the follicular cells from pediatric PTC. In the same line, *RET* fusions in adults are associated with less aggressive tumor behavior and variants of PTC, but in pediatric cases it seems to be associated with distant metastases. *BRAF* fusions, which are highly prevalent in pediatric tumors, are hardly detected in adults. The same is observed for *TERT* promoter mutations. *TERT* promoter mutations are highly associated with older age and a worse prognosis in adults, but are absent or at lower percentages in pediatric PTC.

Remarkable advances in the field of thyroid cancer research have been achieved in recent years, with the development of next generation sequencing (NGS) technologies. NGS is now more accessible to many laboratories and works properly in DNA and RNA isolated from formalin-fixed paraffin-embedded sections, resulting in an unparalleled resolution of genetic and epigenetic events behind cancer initiation and the progression of PTC. The recurrent rearrangements, traditionally identified by methods such as fluorescence in situ hybridization (FISH) and PCR, can be effectively expanded with the use of NGS by finding novel fusion genes and inversions that were challenging to be observed before. We can now not only confirm the relevance of known fusions, but can also identify novel fusion genes in both adult and pediatric PTC cases. In fact, numerous inversions involving chromosome 10 (*RET*) and 7 (*BRAF*) were recently associated with the pathogenesis of PTC.

Although significant progress has been made in thyroid cancer research, we unfortunately cannot extrapolate findings from one cancer to another or even from adult to pediatric PTC. Another major barrier is the spatial (intratumoral) and temporal (primary vs. local or distant metastasis) genetic heterogeneity. Therefore, we still have a knowledge gap in the existing literature. As an example, as the thyroid undergoes important functional changes during aging, it is essential to understand aspects such as (1) how the patient’s lifestyle, immune system, race, ethnicity, and metabolic state influence the fate of a cell; (2) how the immune system’s responses vary with age and gender, thus affecting the range of mutation rates; (3) how many genetic events are required for a cancer driver mutation to convert a normal thyroid cell into a cancer cell in adult and pediatric PTC; (4) whether the described mutations that have been associated with pathogenesis and/or progression of the pediatric and adult PTC could be a direct consequence of genetic instability; (5) whether the selective advantage conferred to follicular cells by a specific cancer driver depend on age and gender; (6) how cells overcome senescence at different ages; and (7) how thyroid-specific cancer driver genes mold the epithelial-to-mesenchymal transition in thyroid-follicular cells and how this changes with aging.

To fill this gap and identify all classes of somatic mutations that confer an advantage on cell clones, as well as timing these mutations during tumor evolution in both adult and pediatric sporadic cases, additional work is still needed. The next step to be taken will likely involve Whole Genome Sequencing (WGS) of different tumor stages. WGS, combined with epidemiologic studies, may also help in identifying the underlying changes that drive cancer phenotypes in adult and pediatric populations. Additionally, we need to go back to the cellular level to better comprehend all molecular findings. It is essential to overcome the limitations of in vitro and in vivo models to truthfully model PTC initiation and progression, as well as epithelial-to-mesenchymal transition at different ages. Furthermore, investigation of key differences in the nuclear architecture of malignant and non-transformed cells is needed to better understand the higher-order structure that regulates transcription and maintains genomic stability.

## Figures and Tables

**Table 1:** Clinical pathological features of pediatric PTC.

Reference	*n*	Distant Met. (%)	LN Met. (%)	Mean Age (y.o.)	Gender F:M	Mean size (cm)	Mean Follow-up (years)	% NED	Mortality
Zimmerman et al. [29]	58	6.9	89.7	< 17	2.2: 1	3.1	26.7	52	14%*
Dottorini et al. [68]	85	18.8	60	14.7	2.86: 1	X	9.25	63.5	0
Kuo et al. [69]	77	18	6.4	12.9	3.3: 1	6.93	8.2	89.6	0
Vaisman et al. [70]	65	29.2	61.5	14	3: 1	2.99	12.6	50.8	0
Fridman et al. [71]	94	20	66	15.1	3: 1	1.2	4.2	97	0
Pires et al. [72]	118	26.9	67.3	13.3	2.6: 1	2.5	8	63.5	0
Cordioli et al. [73]	38	26.3	73.7	11.8	3.2: 1	2.6	7.8	54.1	0
Poyrazoğlu et al. [74]	75	13.3	45.3	12.4	2.1: 1	2.2	4.3	65.3	1 patient
Hampson et al. [75]	62	19.3	46.7	13.8	2.5: 1	2.3	3.6	59.6	Not reported
Galuppini et al. [76]	59	20.8	51	14.4	2.7: 1	2.0	5.9	66.7	Not reported

Mets, metastasis; LN, lymph node; NED, no evidence of disease; * all > 15 y.o.

**Table 2:** Most prevalent genetic alterations described in adult and pediatric PTC.

Genetic Alterations	Adult PTC	Pediatric PTC		
		Sporadic	Post-Chernobyl	Post-Fukushima
*BRAF* ^V600E^	27–83% [100,102,103,104,105,106,107,108]	0–63% [109,110,111,112,113,114,115,116,117,118,119,120,121,122,123]	0–17% [26,117,124,125]	70% [52,126]
*AKAP9-BRAF* fusion	1% [100,124]	0–1% [115,117,124,125]	0–11% [117,124,125]	0% [52]
*AGK-BRAF* fusion	0–0.2% [100,117,127,128]	0–19% [115,117,119,121,129,130,131]	0–4% [117,125,127]	ND
Novel *BRAF* fusions	2% [100]	0–4% [123,131]	10% [125]	ND
*RET/PTC1–3* fusions	5–70% [100,105,132,133]	0–37% [26,87,115,134]	27–77% [87,125,133,135,136,137,138]	6.5% [126]
Other *RET* fusions	1–7% [100,133]	2–7% [115,131]	0–6% [125,139,140,141,142,143,144]	3% [126]
*ETV6-NTRK3* fusion	1–5% [100,127,145]	0–18% [115,117,120,127,131,146]	6–14.5% [125,146,147]	5%[126]
Other *NTRK* fusions	1% [100]	2–4% [115,131]	3% [125]	1.4% [126]
*STRN-ALK* fusion	0–7% [100,127,148]	0–6.5% [123,131,148]	1.4–7% [125,126,139]	1.4% [126]
*PAX8-PPARγ* fusion	0–5% [100,149,150,151,152]	0–9% [113,119,122,123,129]	4% [117]	ND
*RAS* mutations	1–20% [100,105,108,153]	0–7% [111,113,119,120,123,134]	0–9% [26,125]	0% [52]
*TERT* promoter mutation (C250T, C228T)	2–82% [100,108,154,155,156,157]	0–4% [115,131,134,158,159,160]	ND	0% [52]

ND, non-determined.

## References

[1] Ferlay J., Colombet M., Soerjomataram I., Mathers C., Parkin D.M., Piñeros M., Znaor A., Bray F. (2019). Estimating the global cancer incidence and mortality in 2018: GLOBOCAN sources and methods. Int. J. Cancer.

[2] Rahib L., Smith B.D., Aizenberg R., Rosenzweig A.B., Fleshman J.M., Matrisian L.M. (2014). Projecting cancer incidence and deaths to 2030: The unexpected burden of thyroid, liver, and pancreas cancers in the united states. Cancer Res..

[3] Davies L., Welch H.G. (2006). Increasing incidence of thyroid cancer in the United States, 1973–2002. JAMA.

[4] Lise M., Franceschi S., Buzzoni C., Zambon P., Falcini F., Crocetti E., Serraino D., Iachetta F., Zanetti R., Vercelli M. (2012). Changes in the Incidence of Thyroid Cancer Between 1991 and 2005 in Italy: A Geographical Analysis. Thyroid.

[5] Keinan-Boker L., Silverman B.G. (2016). Trends of Thyroid Cancer in Israel: 1980–2012. Rambam Maimonides Med. J..

[6] Lubina A., Cohen O., Barchana M., Liphshiz I., Vered I., Sadetzki S., Karasik A. (2006). Time trends of incidence rates of thyroid cancer in Israel: What might explain the sharp increase. Thyroid.

[7] Wang Y., Wang W. (2015). Increasing incidence of thyroid cancer in Shanghai, China, 1983–2007. Asia-Pac. J. Public Health.

[8] Ahn H.S., Kim H.J., Welch G. (2014). Korea’s Thyroid-Cancer “Epidemic”—Screening and Overdiagnosis. N. Engl. J. Med..

[9] Veiga L.H.S., Neta G., Aschebrook-Kilfoy B., Ron E., Devesa S.S. (2013). Thyroid cancer incidence patterns in Sao Paulo, Brazil, and the U.S. SEER program, 1997–2008. Thyroid.

[10] Sierra M.S., Soerjomataram I., Forman D. (2016). Thyroid cancer burden in Central and South America. Cancer Epidemiol..

[11] Enewold L., Zhu K., Ron E., Marrogi A.J., Stojadinovic A., Peoples G.E., Devesa S.S. (2009). Rising thyroid cancer incidence in the United States by demographic and tumor characteristics, 1980–2005. Cancer Epidemiol. Biomark. Prev..

[12] Kent W.D.T., Hall S.F., Isotalo P.A., Houlden R.L., George R.L., Groome P.A. (2007). Increased incidence of differentiated thyroid carcinoma and detection of subclinical disease. CMAJ.

[13] Liu S., Semenciw R., Ugnat A.M., Mao Y. (2001). Increasing thyroid cancer incidence in Canada, 1970–1996: Time trends and age-period-cohort effects. Br. J. Cancer.

[14] Uhry Z., Colonna M., Remontet L., Grosclaude P., Carré N., Couris C.M., Velten M. (2007). Estimating infra-national and national thyroid cancer incidence in France from cancer registries data and national hospital discharge database. Eur. J. Epidemiol..

[15] Colonna M., Uhry Z., Guizard A.V., Delafosse P., Schvartz C., Belot A., Grosclaude P. (2015). Recent trends in incidence, geographical distribution, and survival of papillary thyroid cancer in France. Cancer Epidemiol..

[16] Reynolds R.M., Weir J., Stockton D.L., Brewster D.H., Sandeep T.C., Strachan M.W.J. (2005). Changing trends in incidence and mortality of thyroid cancer in Scotland. Clin. Endocrinol..

[17] Smailyte G., Miseikyte-Kaubriene E., Kurtinaitis J. (2006). Increasing thyroid cancer incidence in Lithuania in 1978–2003. BMC Cancer.

[18] Pandeya N., McLeod D.S., Balasubramaniam K., Baade P.D., Youl P.H., Bain C.J., Allison R., Jordan S.J. (2016). Increasing thyroid cancer incidence in Queensland, Australia 1982–2008—True increase or overdiagnosis. Clin. Endocrinol..

[19] Howlader N., Noone A., Krapcho M., Miller D., Brest A., Yu M., Ruhl J., Tatalovich Z., Mariotto A., Lewis D. Cancer Statistics Review, 1975–2017—SEER Statistics. https://seer.cancer.gov/csr/1975_2017/.

[20] Howlader N., Noone A., Krapcho M., Miller D., Brest A., Yu M., Ruhl J., Tatalovich Z., Mariotto A., Lewis D. SEER Cancer Statistics Review, 1975–2016. https://seer.cancer.gov/csr/1975_2016/.

[21] Francis G.L., Waguespack S.G., Bauer A.J., Angelos P., Benvenga S., Cerutti J.M., Dinauer C.A., Hamilton J., Hay I.D., Luster M. (2015). Management Guidelines for Children with Thyroid Nodules and Differentiated Thyroid Cancer. Thyroid.

[22] Jarząb B., Handkiewicz-Junak D., Włoch J. (2005). Juvenile differentiated thyroid carcinoma and the role of radioiodine in its treatment: A qualitative review. Endocr. Relat. Cancer.

[23] Hogan A., Zhuge Y., Perez E., Koniaris L., Lew J., Sola J. (2009). The incidence of pediatric thyroid cancer is increasing and is higher in girls than in boys and may have an adverse outcome. Clin. Thyroidol..

[24] Vaisman F., Corbo R., Vaisman M. (2011). Thyroid Carcinoma in Children and Adolescents—Systematic Review of the Literature. J. Thyroid Res..

[25] Wu X.-C., Chen V.W., Steele B., Roffers S., Klotz J.B., Correa C.N., Carozza S.E. (2003). Cancer incidence in adolescents and young adults in the United States, 1992–1997. J. Adolesc. Health.

[26] Cordioli M.I., Moraes L., Cury A.N., Cerutti J.M. (2015). Are we really at the dawn of understanding sporadic pediatric thyroid carcinoma?. Endocr. Relat. Cancer.

[27] Tuttle R.M., Ball D.W., Byrd D., Dilawari R.A., Gerard M., Duh Q., Ehya H., Farrar W.B., Haddad R.I., Kandeel F. (2010). Thyroid Carcinoma. J. Natl. Compr. Cancer Netw..

[28] Chan C.M., Young J., Prager J., Travers S. (2017). Pediatric Thyroid Cancer. Adv. Pediatr..

[29] Zimmerman D., Hay I.D., Gough I.R., Goellner J.R., Ryan J.J., Grant C.S., McConahey W.M. (1988). Papillary thyroid carcinoma in children and adults: Long-term follow-up of 1039 patients conservatively treated at one institution during three decades. Surgery.

[30] Haugen B.R., Alexander E.K., Bible K.C., Doherty G.M., Mandel S.J., Nikiforov Y.E., Pacini F., Randolph G.W., Sawka A.M., Schlumberger M. (2016). 2015 American Thyroid Association Management Guidelines for Adult Patients with Thyroid Nodules and Differentiated Thyroid Cancer: The American Thyroid Association Guidelines Task Force on Thyroid Nodules and Differentiated Thyroid Cancer. Thyroid.

[31] Karapanou O., Tzanela M., Vlassopoulou B., Kanaka-Gantenbein C. (2017). Differentiated thyroid cancer in childhood: A literature update. Hormones.

[32] Creo A., Alahdab F., Al Nofal A., Thomas K., Kolbe A., Pittock S.T. (2018). Ultrasonography and the American Thyroid Association Ultrasound-Based Risk Stratification Tool: Utility in Pediatric and Adolescent Thyroid Nodules. Horm. Res. Paediatr..

[33] Hay I.D., Gonzalez-Losada T., Reinalda M.S., Honetschlager J.A., Richards M.L., Thompson G.B. (2010). Long-term outcome in 215 children and adolescents with papillary thyroid cancer treated during 1940 through 2008. World J. Surg..

[34] Zaydfudim V., Feurer I.D., Griffin M.R., Phay J.E. (2008). The impact of lymph node involvement on survival in patients with papillary and follicular thyroid carcinoma. Surgery.

[35] Ahn B.H., Kim J.R., Jeong H.C., Lee J.S., Chang E.S., Kim Y.H. (2015). Predictive factors of central lymph node metastasis in papillary thyroid carcinoma. Ann. Surg. Treat. Res..

[36] Pawelczak M., David R., Franklin B., Kessler M., Lam L., Shah B. (2010). Outcomes of children and adolescents with well-differentiated thyroid carcinoma and pulmonary metastases following 131I treatment: A systematic review. Thyroid.

[37] Handkiewicz-Junak D., Wloch J., Roskosz J., Krajewska J., Kropinska A., Pomorski L., Kukulska A., Prokurat A., Wygoda Z., Jarzab B. (2007). Total thyroidectomy and adjuvant radioiodine treatment independently decrease locoregional recurrence risk in childhood and adolescent differentiated thyroid cancer. J. Nucl. Med..

[38] Brito J.P., Davies L. (2014). Is there really an increased incidence of thyroid cancer?. Curr. Opin. Endocrinol. Diabetes Obes..

[39] Franceschi S., Vaccarella S., La Vecchia C., Bosetti C., Malvezzi M., Garavello W., Bertuccio P., Levi F., Negri E. (2015). Thyroid cancer: An epidemic of disease or an epidemic of diagnosis?. Int. J. Cancer.

[40] Morris L.G., Tuttle R.M., Davies L. (2016). Changing Trends in the Incidence of Thyroid Cancer in the United States. JAMA Otolaryngol. Head Neck Surg..

[41] Sholl L.M., Barletta J.A., Hornick J.L. (2017). Radiation-associated neoplasia: Clinical, pathological and genomic correlates. Histopathology.

[42] Cléro É., Doyon F., Chungue V., Rachédi F., Boissin J.-L., Sebbag J., Shan L., Bost-Bezeaud F., Petitdidier P., Dewailly É. (2012). Dietary Iodine and Thyroid Cancer Risk in French Polynesia: A Case—Control Study. Thyroid.

[43] Engeland A., Tretli S., Akslen L.A., Bjørge T. (2006). Body size and thyroid cancer in two million Norwegian men and women. Br. J. Cancer.

[44] Vigneri R., Malandrino P., Gianì F., Russo M., Vigneri P. (2016). Heavy metals in the volcanic environment and thyroid cancer. Mol. Cell. Endocrinol..

[45] Ron E., Lubin J.H., Shore R.E., Mabuchi K., Modan B., Pottern L.M., Schneider A.B., Tucker M.A., Boice J.D. (1995). Thyroid cancer after exposure to external radiation: A pooled analysis of seven studies. Radiat. Res..

[46] Sadetzki S., Chetrit A., Lubina A., Stovall M., Novikov I. (2006). Risk of thyroid cancer after childhood exposure to ionizing radiation for tinea capitis. J. Clin. Endocrinol. Metab..

[47] Goldschmidt H. (1977). Dermatologic Radiotherapy and Thyroid Cancer. Arch. Dermatol..

[48] Wijnen M., van den Heuvel-Eibrink M.M., Medici M., Peeters R.P., van der Lely A.J., Neggers S.J. (2016). Risk factors for subsequent endocrine-related cancer in childhood cancer survivors. Endocr. Relat. Cancer.

[49] Ishikawa T. (2017). Radiation Doses and Associated Risk from the Fukushima Nuclear Accident. Asia Pac. J. Public Health.

[50] Yamamoto H., Hayashi K., Scherb H., Efird J.T. (2019). Association between the detection rate of thyroid cancer and the external radiation dose-rate after the nuclear power plant accidents in Fukushima, Japan. Medicine.

[51] Toki H., Wada T., Manabe Y., Hirota S., Higuchi T., Tanihata I., Satoh K., Bando M. (2020). Relationship between environmental radiation and radioactivity and childhood thyroid cancer found in Fukushima health management survey. Sci. Rep..

[52] Mitsutake N., Fukushima T., Matsuse M., Rogounovitch T., Saenko V., Uchino S., Ito M., Suzuki K., Suzuki S., Yamashita S. (2015). BRAFV600E mutation is highly prevalent in thyroid carcinomas in the young population in Fukushima: A different oncogenic profile from Chernobyl. Sci. Rep..

[53] Yamashita S., Suzuki S., Suzuki S., Shimura H., Saenko V. (2018). Lessons from Fukushima: Latest Findings of Thyroid Cancer after the Fukushima Nuclear Power Plant Accident. Thyroid.

[54] Alzahrani A.S., Alkhafaji D., Tuli M., Al-Hindi H., Sadiq B. (2015). Bin Comparison of differentiated thyroid cancer in children and adolescents (≤20 years) with young adults. Clin. Endocrinol..

[55] Lee Y.A., Jung H.W., Kim H.Y., Choi H., Kim H.-Y., Hah J.H., Park D.J., Chung J.-K., Yang S.W., Shin C.H. (2015). Pediatric Patients with Multifocal Papillary Thyroid Cancer Have Higher Recurrence Rates than Adult Patients: A Retrospective Analysis of a Large Pediatric Thyroid Cancer Cohort over 33 Years. J. Clin. Endocrinol. Metab..

[56] Hay I.D., Johnson T.R., Kaggal S., Reinalda M.S., Iniguez-Ariza N.M., Grant C.S., Pittock S.T., Thompson G.B. (2018). Papillary Thyroid Carcinoma (PTC) in Children and Adults: Comparison of Initial Presentation and Long-Term Postoperative Outcome in 4432 Patients Consecutively Treated at the Mayo Clinic during Eight Decades (1936–2015). World J. Surg..

[57] Hogan A.R., Zhuge Y., Perez E.A., Koniaris L.G., Lew J.I., Sola J.E. (2009). Pediatric thyroid carcinoma: Incidence and outcomes in 1753 patients. J. Surg. Res..

[58] de Jong M.C., Gaze M.N., Szychot E., Rozalén García V., Brain C., Dattani M., Spoudeas H., Hindmarsh P., Abdel-Aziz T.E., Bomanji J. (2020). Treating papillary and follicular thyroid cancer in children and young people: Single UK-center experience between 2003 and 2018. J. Pediatr. Surg..

[59] Rah C.S., Kim W.W., Lee Y.M., Kim W.G., Song D.E., Chung K.W., Kim S.C., Hong S.J., Sung T.Y. (2019). Recent Trends in the Clinicopathological Features of Thyroid Nodules in Pediatric Patients: A Single Tertiary Center Experience over 25 Years. Int. J. Endocrinol..

[60] Niedziela M. (2006). Pathogenesis, diagnosis and management of thyroid nodules in children. Endocr. Relat. Cancer.

[61] Durante C., Grani G., Lamartina L., Filetti S., Mandel S.J., Cooper D.S. (2018). The diagnosis and management of thyroid nodules a review. JAMA J. Am. Med. Assoc..

[62] Davies L., Welch H.G. (2014). Current thyroid cancer trends in the United States. JAMA Otolaryngol. Head Neck Surg..

[63] Lerner J., Goldfarb M. (2015). Pediatric Thyroid Microcarcinoma. Ann. Surg. Oncol..

[64] Bernier M.O., Withrow D.R., Berrington de Gonzalez A., Lam C.J.K., Linet M.S., Kitahara C.M., Shiels M.S. (2019). Trends in pediatric thyroid cancer incidence in the United States, 1998–2013. Cancer.

[65] Golpanian S., Perez E.A., Tashiro J., Lew J.I., Sola J.E., Hogan A.R. (2016). Pediatric papillary thyroid carcinoma: Outcomes and survival predictors in 2504 surgical patients. Pediatr. Surg. Int..

[66] Chow S.M., Law S.C., Mendenhall W.M., Au S.K., Yau S., Mang O., Lau W.H. (2004). Differentiated thyroid carcinoma in childhood and adolescence-clinical course and role of radioiodine. Pediatr. Blood Cancer.

[67] Liu Z., Hu D., Huang Y., Chen S., Zeng W., Zhou L., Zhou W., Wang M., Feng H., Wei W. (2019). Factors associated with distant metastasis in pediatric thyroid cancer: Evaluation of the seer database. Endocr. Connect..

[68] Dottorini M.E., Vignati A., Mazzucchelli L., Lomuscio G., Colombo L. (1997). Differentiated thyroid carcinoma in children and adolescents: A 37-year experience in 85 patients. J. Nucl. Med..

[69] Kuo S.-F., Chao T.-C., Hsueh C., Chuang W.-Y., Yang C.-H., Lin J.-D. (2008). Prognosis and Risk Stratification in Young Papillary Thyroid Carcinoma Patients. Endocr. J..

[70] Vaisman F., Bulzico D.A., Pessoa C.H., Bordallo M.A., de Mendonça U.B., Dias F.L., Coeli C.M., Corbo R., Vaisman M. (2011). Prognostic factors of a good response to initial therapy in children and adolescents with differentiated thyroid cancer. Clinics.

[71] Fridman M.V., Savva N.N., Krasko O.V., Zborovskaya A.A., Mankovskaya S.V., Schmid K.W., Demidchik Y.E. (2012). Clinical and Pathologic Features of “Sporadic” Papillary Thyroid Carcinoma Registered in the Years 2005 to 2008 in Children and Adolescents of Belarus. Thyroid.

[72] Pires B.P., Alves P.A.G., Bordallo M.A., Bulzico D.A., Lopes F.P., Farias T., Dias F., Lima R.A., Santos Gisler I.C., Coeli C.M. (2016). Prognostic Factors for Early and Long-Term Remission in Pediatric Differentiated Thyroid Carcinoma: The Role of Sex, Age, Clinical Presentation, and the Newly Proposed American Thyroid Association Risk Stratification System. Thyroid.

[73] Cordioli M.I., Moraes L., Alves M.T., Delcelo R., Monte O., Longui C.A., Cury A.N., Cerutti J.M. (2016). Thyroid-specific genes expression uncovered age-related differences in pediatric thyroid carcinomas. Int. J. Endocrinol..

[74] Poyrazoğlu Ş., Bundak R., Baş F., Yeğen G., Şanlı Y., Darendeliler F. (2017). Clinicopathological characteristics of papillary thyroid cancer in children with emphasis on pubertal status and association with BRAFV600E mutation. JCRPE J. Clin. Res. Pediatr. Endocrinol..

[75] Hampson S., Stephens D., Wasserman J.D. (2018). Young age is associated with increased rates of residual and recurrent paediatric differentiated thyroid carcinoma. Clin. Endocrinol..

[76] Galuppini F., Vianello F., Censi S., Barollo S., Bertazza L., Carducci S., Colato C., Manso J., Rugge M., Iacobone M. (2019). Differentiated Thyroid Carcinoma in Pediatric Age: Genetic and Clinical Scenario. Front. Endocrinol..

[77] Mazzaferri E.L., Massoll N. (2002). Management of papillary and follicular (differentiated) thyroid cancer: New paradigms using recombinant human thyrotropin. Endocr. Relat. Cancer.

[78] Mazzaferri E.L., Kloos R.T. (2001). Clinical review 128: Current approaches to primary therapy for papillary and follicular thyroid cancer. J. Clin. Endocrinol. Metab..

[79] Demidchik Y.E., Demidchik E.P., Reiners C., Biko J., Mine M., Saenko V.A., Yamashita S. (2006). Comprehensive clinical assessment of 740 cases of surgically treated thyroid cancer in children of Belarus. Ann. Surg..

[80] Lazar L., Lebenthal Y., Steinmetz A., Yackobovitch-Gavan M., Phillip M. (2009). Differentiated Thyroid Carcinoma in Pediatric Patients: Comparison of Presentation and Course between Pre-Pubertal Children and Adolescents. J. Pediatr..

[81] Van Santen H.M., Aronson D.C., Vulsma T., Tummers R.F., Geenen M.M., De Vijlder J.J., Van Den Bos C. (2004). Frequent adverse events after treatment for childhood-onset differentiated thyroid carcinoma: A single institute experience. Eur. J. Cancer.

[82] Schneider R., Reiners C. (2003). The Effect of Levothyroxine Therapy on Bone Mineral Density: A Systematic Review of the Literature. Exp. Clin. Endocrinol. Diabetes.

[83] Fridman M., Krasko O., Branovan D.I., Dabryian S., Pisarenko A., Lo C.Y., Lam A.K. (2019). yin Factors affecting the approaches and complications of surgery in childhood papillary thyroid carcinomas. Eur. J. Surg. Oncol..

[84] Maxon H.R. (1999). Quantitative radioiodine therapy in the treatment of differentiated thyroid cancer. Q. J. Nucl. Med..

[85] Zidan J., Hefer E., Iosilevski G., Drumea K., Stein M.E., Kuten A., Israel O. (2004). Efficacy of i131 ablation therapy using different doses as determined by postoperative thyroid scan uptake in patients with differentiated thyroid cancer. Int. J. Radiat. Oncol. Biol. Phys..

[86] Haddad R.I., Nasr C., Bischoff L., Busaidy N.L., Byrd D., Callender G., Dickson P., Duh Q.Y., Ehya H., Goldner W. (2018). Thyroid carcinoma, version 2.2018 featured updates to the NCCN guidelines. JNCCN J. Natl. Compr. Cancer Netw..

[87] Paulson V.A., Rudzinski E.R., Hawkins D.S. (2019). Thyroid cancer in the pediatric population. Genes.

[88] Van Wyngaarden M., McDougall I.R. (1996). What is the role of 1100 MBq (<30 mCi) radioiodine 131I in the treatment of patients with differentiated thyroid cancer?. Nucl. Med. Commun..

[89] Pacini F., Schlumberger M., Dralle H., Elisei R., Smit J.W.A., Wiersinga W., Moreno-Reyes R., Van den Bruel A., Zira C., Feldt-Rasmussen U. (2006). European consensus for the management of patients with differentiated thyroid carcinoma of the follicular epithelium. Eur. J. Endocrinol..

[90] Mazzaferri E.L., Jhiang S.M. (1994). Long-term impact of initial surgical and medical therapy on papillary and follicular thyroid cancer. Am. J. Med..

[91] Schlumberger M., Pacini F., Wiersinga W.M., Toft A., Smit J.W.A., Franco F.S., Lind P., Limbert E., Jarzab B., Jamar F. (2004). Follow-up and management of differentiated thyroid carcinoma: A European perspective in clinical practice. Eur. J. Endocrinol..

[92] Biondi B., Fazio S., Carella C., Amato G., Cittadini A., Lupoli G., Saccà L., Bellastella A., Lombardi G. (1993). Cardiac effects of long term thyrotropin-suppressive therapy with levothyroxine. J. Clin. Endocrinol. Metab..

[93] Matuszewska G., Roskosz J., Wloch J., Jurecka-Tuleja B., Hasse-Lazar K., Kowalczyk B., Jarzab B. (2001). Evaluation of effects of L-thyroxine therapy in differentiated thyroid carcinoma on the cardiovascular system—Prospective study. Wiad. Lek..

[94] Chow S.M., Yau S., Lee S.H., Leung W.M., Law S.C.K. (2004). Pregnancy outcome after diagnosis of differentiated thyroid carcinoma: No deleterious effect after radioactive iodine treatment. Int. J. Radiat. Oncol. Biol. Phys..

[95] Krassas G.E., Pontikides N. (2005). Gonadal effect of radiation from 131I in male patients with thyroid carcinoma. Arch. Androl..

[96] Wichers M., Benz E., Palmedo H., Biersack H.J., Grünwald F., Klingmüller D. (2000). Testicular function after radioiodine therapy for thyroid carcinoma. Eur. J. Nucl. Med..

[97] Nikiforov Y. (2011). Molecular diagnosis of thyroid tumors. Arch. Pathol. Lab. Med..

[98] Xing M., Haugen B.R., Schlumberger M. (2013). Progress in molecular-based management of differentiated thyroid cancer. Lancet.

[99] Xing M. (2013). Molecular pathogenesis and mechanisms of thyroid cancer. Nat. Rev. Cancer.

[100] Agrawal N., Akbani R., Aksoy B.A., Ally A., Arachchi H., Asa S.L., Auman J.T., Balasundaram M., Balu S., Baylin S.B. (2014). Integrated Genomic Characterization of Papillary Thyroid Carcinoma. Cell.

[101] Liu T., Wang N., Cao J., Sofiadis A., Dinets A., Zedenius J., Larsson C., Xu D. (2014). The age-and shorter telomere-dependent tert promoter mutation in follicular thyroid cell-derived carcinomas. Oncogene.

[102] Kimura E.T., Nikiforova M.N., Zhu Z., Knauf J.A., Nikiforov Y.E., Fagin J.A. (2003). High Prevalence of BRAF Mutations in Thyroid Cancer: Genetic Evidence for Constitutive Activation of the RET/PTC-RAS-BRAF Signaling Pathway in Papillary Thyroid Carcinoma. Cancer Res..

[103] Cohen Y., Xing M., Mambo E., Gou Z., Wu G., Trink B., Beller U., Westra W.H., Ladenson P.W., Sidransky D. (2003). BRAF mutation in papillary thyroid carcinoma. J. Natl. Cancer Inst..

[104] Xing M. (2005). BRAF mutation in thyroid cancer. Endocr. Relat. Cancer.

[105] Bastos A.U., Oler G., Nozima B.H.N., Moysés R.A., Cerutti J.M. (2015). BRAF V600E and decreased NIS and TPO expression are associated with aggressiveness of a subgroup of papillary thyroid microcarcinoma. Eur. J. Endocrinol..

[106] Oler G., Cerutti J.M. (2009). High prevalence of BRAF mutation in a Brazilian cohort of patients with sporadic papillary thyroid carcinomas. Cancer.

[107] Al-Salam S., Sharma C., Afandi B., Al Dahmani K., Al-Zahrani A.S., Al Shamsi A., Al Kaabi J. (2020). BRAF and KRAS mutations in papillary thyroid carcinoma in the United Arab Emirates. PLoS ONE.

[108] Rusinek D., Pfeifer A., Cieslicka M., Kowalska M., Pawlaczek A., Krajewska J., Szpak-Ulczok S., Tyszkiewicz T., Halczok M., Czarniecka A. (2020). TERT Promoter Mutations and Their Impact on Gene Expression Profile in Papillary Thyroid Carcinoma. Cancers.

[109] Lima J., Trovisco V., Soares P., Máximo V., Magalhães J., Salvatore G., Santoro M., Bogdanova T., Tronko M., Abrosimov A. (2004). BRAF mutations are not a major event in post-chernobyl childhood thyroid carcinomas. J. Clin. Endocrinol. Metab..

[110] Kumagai A., Namba H., Saenko V.A., Ashizawa K., Ohtsuru A., Ito M., Ishikawa N., Sugino K., Ito K., Jeremiah S. (2004). Low frequency of BRAFT1796A mutations in childhood thyroid carcinomas. J. Clin. Endocrinol. Metab..

[111] Alzahrani A.S., Murugan A.K., Qasem E., Alswailem M., Al-Hindi H., Shi Y. (2017). Single Point Mutations in Pediatric Differentiated Thyroid Cancer. Thyroid.

[112] Geng J., Wang H., Liu Y., Tai J., Jin Y., Zhang J., He L., Fu L., Qin H., Song Y. (2017). Correlation between BRAFV600E mutation and clinicopathological features in pediatric papillary thyroid carcinoma. Sci. China Life Sci..

[113] Mostoufi-Moab S., Labourier E., Sullivan L., LiVolsi V., Li Y., Xiao R., Beaudenon-Huibregtse S., Kazahaya K., Adzick N.S., Baloch Z. (2018). Molecular Testing for Oncogenic Gene Alterations in Pediatric Thyroid Lesions. Thyroid.

[114] Henke L.E., Perkins S.M., Pfeifer J.D., Ma C., Chen Y., Dewees T., Grigsby P.W. (2014). BRAF V600E mutational status in pediatric thyroid cancer. Pediatr. Blood Cancer.

[115] Alzahrani A.S., Alswailem M., Alswailem A.A., Al-Hindi H., Goljan E., Alsudairy N., Abouelhoda M. (2020). Genetic Alterations in Pediatric Thyroid Cancer Using a Comprehensive Childhood Cancer Gene Panel. J. Clin. Endocrinol. Metab..

[116] Rosenbaum E., Hosler G., Zahurak M., Cohen Y., Sidransky D., Westra W.H. (2005). Mutational activation of BRAF is not a major event in sporadic childhood papillary thyroid carcinoma. Mod. Pathol..

[117] Ricarte-Filho J.C., Li S., Garcia-Rendueles M.E.R., Montero-Conde C., Voza F., Knauf J.A., Heguy A., Viale A., Bogdanova T., Thomas G.A. (2013). Identification of kinase fusion oncogenes in post-Chernobyl radiation-induced thyroid cancers. J. Clin. Investig..

[118] Givens D.J., Buchmann L.O., Agarwal A.M., Grimmer J.F., Hunt J.P. (2014). BRAF V600E does not predict aggressive features of pediatric papillary thyroid carcinoma. Laryngoscope.

[119] Prasad M.L., Vyas M., Horne M.J., Virk R.K., Morotti R., Liu Z., Tallini G., Nikiforova M.N., Christison-Lagay E.R., Udelsman R. (2016). NTRK fusion oncogenes in pediatric papillary thyroid carcinoma in northeast United States. Cancer.

[120] Cordioli M.I., Moraes L., Bastos A.U., Besson P., Alves M.T., Delcelo R., Monte O., Longui C., Cury A.N., Cerutti J.M. (2017). Fusion Oncogenes Are the Main Genetic Events Found in Sporadic Papillary Thyroid Carcinomas from Children. Thyroid.

[121] Sisdelli L., Cordioli M.I., Vaisman F., Moraes L., Colozza-Gama G.A., Alves P.A.G., Araújo M.L., Alves M.T.S., Monte O., Longui C.A. (2019). AGK-BRAF is associated with distant metastasis and younger age in pediatric papillary thyroid carcinoma. Pediatr. Blood Cancer.

[122] Nikita M.E., Jiang W., Cheng S.-M., Hantash F.M., McPhaul M.J., Newbury R.O., Phillips S.A., Reitz R.E., Waldman F.M., Newfield R.S. (2016). Mutational Analysis in Pediatric Thyroid Cancer and Correlations with Age, Ethnicity, and Clinical Presentation. Thyroid.

[123] Gertz R.J., Nikiforov Y., Rehrauer W., McDaniel L., Lloyd R.V. (2016). Mutation in BRAF and Other Members of the MAPK Pathway in Papillary Thyroid Carcinoma in the Pediatric Population. Arch. Pathol. Lab. Med..

[124] Ciampi R., Knauf J.A., Kerler R., Gandhi M., Zhu Z., Nikiforova M.N., Rabes H.M., Fagin J.A., Nikiforov Y.E. (2005). Oncogenic AKAP9-BRAF fusion is a novel mechanism of MAPK pathway activation in thyroid cancer. J. Clin. Investig..

[125] Efanov A.A., Brenner A.V., Bogdanova T.I., Kelly L.M., Liu P., Little M.P., Wald A.I., Hatch M., Zurnadzy L.Y., Nikiforova M.N. (2018). Investigation of the relationship between radiation dose and gene mutations and fusions in post-chernobyl thyroid cancer. J. Natl. Cancer Inst..

[126] Iwadate M., Mitsutake N., Matsuse M., Fukushima T., Suzuki S., Matsumoto Y., Ookouchi C., Mizunuma H., Nakamura I., Nakano K. (2020). The clinicopathological results of thyroid cancer with BRAFV600E mutation in the young population of Fukushima. J. Clin. Endocrinol. Metab..

[127] Bastos A.U., de Jesus A.C., Cerutti J.M. (2018). ETV6-NTRK3 and STRN-ALK kinase fusions are recurrent events in papillary thyroid cancer of adult population. Eur. J. Endocrinol..

[128] Ross J.S., Wang K., Chmielecki J., Gay L., Johnson A., Chudnovsky J., Yelensky R., Lipson D., Ali S.M., Elvin J.A. (2015). The distribution of BRAF gene fusions in solid tumors and response to targeted therapy. Int. J. Cancer.

[129] Picarsic J.L., Buryk M.A., Ozolek J., Ranganathan S., Monaco S.E., Simons J.P., Witchel S.F., Gurtunca N., Joyce J., Zhong S. (2016). Molecular Characterization of Sporadic Pediatric Thyroid Carcinoma with the DNA/RNA ThyroSeq v2 Next-Generation Sequencing Assay. Pediatr. Dev. Pathol..

[130] Vanden Borre P., Schrock A.B., Anderson P.M., Morris J.C., Heilmann A.M., Holmes O., Wang K., Johnson A., Waguespack S.G., Ou S.I. (2017). Pediatric, Adolescent, and Young Adult Thyroid Carcinoma Harbors Frequent and Diverse Targetable Genomic Alterations, Including Kinase Fusions. Oncologist.

[131] Pekova B., Sykorova V., Dvorakova S., Vaclavikova E., Moravcova J., Katra R., Astl J., Vlcek P., Kodetova D., Vcelak J. (2020). RET, NTRK, ALK, BRAF and MET fusions in a large cohort of pediatric papillary thyroid carcinomas. Thyroid.

[132] Santoro M., Carlomagno F. (2013). Central Role of RET in Thyroid Cancer. Cold Spring Harb. Perspect. Biol..

[133] Santoro M., Moccia M., Federico G., Carlomagno F. (2020). Ret gene fusions in malignancies of the thyroid and other tissues. Genes.

[134] Pekova B., Dvorakova S., Sykorova V., Vacinova G., Vaclavikova E., Moravcova J., Katra R., Vlcek P., Sykorova P., Kodetova D. (2019). Somatic genetic alterations in a large cohort of pediatric thyroid nodules. Endocr. Connect..

[135] Pisarchik A., Ermak G., Demidchik E., Mikhalevich L., Kartel N., Figge J. (1998). Low prevalence of the ret/PTC3r1 rearrangement in a series of papillary thyroid carcinomas presenting in Belarus ten years post-Chernobyl. Thyroid.

[136] Unger K., Zitzelsberger H., Salvatore G., Santoro M., Bogdanova T., Braselmann H., Kastner P., Zurnadzhy L., Tronko N., Hutzler P. (2004). Heterogeneity in the distribution of RET/PTC rearrangements within individual post-chernobyl papillary thyroid carcinomas. J. Clin. Endocrinol. Metab..

[137] Nikiforov Y.E., Rowland J.M., Bove K.E., Monforte-Munoz H., Fagin J.A. (1997). Distinct pattern of ret oncogene rearrangements in morphological variants of radiation-induced and sporadic thyroid papillary carcinomas in children. Cancer Res..

[138] Kjellman P., Learoyd D.L., Messina M., Weber G., Höög A., Wallin G., Larsson C., Robinson B.G., Zedenius J. (2001). Expression of the *RET* proto-oncogene in papillary thyroid carcinoma and its correlation with clinical outcome. Br. J. Surg..

[139] Iyama K., Matsuse M., Mitsutake N., Rogounovitch T., Saenko V.A., Suzuki K., Ashizawa M., Ookouchi C., Suzuki S.S.S., Mizunuma H. (2017). Identification of Three Novel Fusion Oncogenes, SQSTM1/NTRK3, AFAP1L2/RET, and PPFIBP2/RET in Thyroid Cancers of Young Patients in Fukushima. Thyroid.

[140] Hamatani K., Eguchi H., Koyama K., Mukai M., Nakachi K., Kusunoki Y. (2014). A novel RET rearrangement (ACBD5/RET) by pericentric inversion, inv(10)(p12.1;q11.2), in papillary thyroid cancer from an atomic bomb survivor exposed to high-dose radiation. Oncol. Rep..

[141] Klugbauer S., Demidchik E.P., Lengfelder E., Rabes H.M. (1998). Detection of a novel type of RET rearrangement (PTC5) in thyroid carcinomas after chernobyl and analysis of the involved RET-fused gene RFG5. Cancer Res..

[142] Klugbauer S., Jauch A., Lengfelder E., Demidchik E., Rabes H.M. (2000). A novel type of RET rearrangement (PTC8) in childhood papillary thyroid carcinomas and characterization of the involved gene (RFG8). Cancer Res..

[143] Salassidis K., Bruch J., Zitzelsberger H., Lengfelder E., Kellerer A.M., Bauchinger M. (2000). Translocation t(10;14)(q11.2;q22.1) fusing the kinectin to the RET gene creates a novel rearranged form (PTC8) of the RET proto-oncogene in radiation-induced childhood papillary thyroid carcinoma. Cancer Res..

[144] Fugazzola L., Pierotti M., Vigano E., Pacini F., Vorontsova T., Bongarzone I. (1996). Molecular and biochemical analysis of RET/PTC4, a novel oncogenic rearrangement between RET and ELE1 genes, in a post-Chernobyl papillary thyroid cancer. Oncogene.

[145] Yoo S.-K., Lee S., Kim S., Jee H.-G., Kim B.-A., Cho H., Song Y.S., Cho S.W., Won J.-K., Shin J.-Y. (2016). Comprehensive Analysis of the Transcriptional and Mutational Landscape of Follicular and Papillary Thyroid Cancers. PLoS Genet..

[146] Leeman-Neill R.J., Kelly L.M., Liu P., Brenner A.V., Little M.P., Bogdanova T.I., Evdokimova V.N., Hatch M., Zurnadzy L.Y., Nikiforova M.N. (2013). ETV6-NTRK3 is a common chromosomal rearrangement in radiation-associated thyroid cancer. Cancer.

[147] Santoro M., Carlomagno F. (2013). Oncogenic rearrangements driving ionizing radiation-associated human cancer. J. Clin. Investig..

[148] Kelly L.M., Barila G., Liu P., Evdokimova V.N., Trivedi S., Panebianco F., Gandhi M., Carty S.E., Hodak S.P., Luo J. (2014). Identification of the transforming STRN-ALK fusion asa potential therapeutic target in the aggressive forms of thyroid cancer. Proc. Natl. Acad. Sci. USA.

[149] Castro P., Rebocho A.P., Soares R.J., Magalhães J., Roque L., Trovisco V., Vieira De Castro I., Cardoso-De-Oliveira M., Fonseca E., Soares P. (2006). PAX8-PPARγ rearrangement is frequently detected in the follicular variant of papillary thyroid carcinoma. J. Clin. Endocrinol. Metab..

[150] Nikiforova M.N., Biddinger P.W., Caudill C.M., Kroll T.G., Nikiforov Y.E. (2002). PAX8-PPARγ rearrangement in thyroid tumors: RT-PCR and immunohistochemical analyses. Am. J. Surg. Pathol..

[151] French C.A., Alexander E.K., Cibas E.S., Nose V., Laguette J., Faquin W., Garber J., Moore F., Fletcher J.A., Larsen P.R. (2003). Genetic and biological subgroups of low-stage follicular thyroid cancer. Am. J. Pathol..

[152] de Jesus Paniza A.C., Mendes T.B., Viana M.D., Thomaz D.M., Chiappini P., Colozza-Gama G.A., Lindsey S.C., de Carvalho M.B., Alves V.A., Curioni O. (2019). Revised criteria for diagnosis of NIFTP reveals a better correlation with tumor biological behavior. Endocr. Connect..

[153] Nikiforov Y.E., Nikiforova M.N. (2011). Molecular genetics and diagnosis of thyroid cancer. Nat. Rev. Endocrinol..

[154] Melo M., Gaspar da Rocha A., Batista R., Vinagre J., Martins M.J., Costa G., Ribeiro C., Carrilho F., Leite V., Lobo C. (2017). TERT, BRAF, and NRAS in Primary Thyroid Cancer and Metastatic Disease. J. Clin. Endocrinol. Metab..

[155] Liu R., Xing M. (2016). TERT promoter mutations in thyroid cancer. Endocr. Relat. Cancer.

[156] Panebianco F., Nikitski A.V., Nikiforova M.N., Nikiforov Y.E. (2019). Spectrum of *TERT* promoter mutations and mechanisms of activation in thyroid cancer. Cancer Med..

[157] Yang J., Gong Y., Yan S., Chen H., Qin S., Gong R. (2020). Association between TERT promoter mutations and clinical behaviors in differentiated thyroid carcinoma: A systematic review and meta-analysis. Endocrine.

[158] Onder S., Ozturk Sari S., Yegen G., Sormaz I.C., Yilmaz I., Poyrazoglu S., Sanlı Y., Giles Senyurek Y., Kapran Y., Mete O. (2016). Classic Architecture with Multicentricity and Local Recurrence, and Absence of TERT Promoter Mutations are Correlates of BRAF V600E Harboring Pediatric Papillary Thyroid Carcinomas. Endocr. Pathol..

[159] Alzahrani A.S., Qasem E., Murugan A.K., Al-Hindi H.N., AlKhafaji D., Almohanna M., Xing M., Alhomaidah D., AlSwailem M. (2016). Uncommon *TERT* Promoter Mutations in Pediatric Thyroid Cancer. Thyroid.

[160] Oishi N., Kondo T., Nakazawa T., Mochizuki K., Inoue T., Kasai K., Tahara I., Yabuta T., Hirokawa M., Miyauchi A. (2017). Frequent BRAF V600E and Absence of TERT Promoter Mutations Characterize Sporadic Pediatric Papillary Thyroid Carcinomas in Japan. Endocr. Pathol..

[161] Cordioli M.I., Moraes L., Carvalheira G., Sisdelli L., Alves M.T., Delcelo R., Monte O., Longui C.A., Cury A.N., Cerutti J.M. (2016). AGK-BRAF gene fusion is a recurrent event in sporadic pediatric thyroid carcinoma. Cancer Med..

[162] Nikiforov Y.E., Seethala R.R., Tallini G., Baloch Z.W., Basolo F., Thompson L.D.R., Barletta J.A., Wenig B.M., Ghuzlan A.A., Kakudo K. (2016). Nomenclature revision for encapsulated follicular variant of papillary thyroid carcinoma a paradigm shift to reduce overtreatment of indolent tumors. JAMA Oncol..

[163] Chu Y.H., Sadow P.M. (2020). Noninvasive follicular thyroid neoplasm with papillary-like nuclear features (NIFTP): Diagnostic updates and molecular advances. Semin. Diagn. Pathol..

[164] Wan P.T., Garnett M.J., Roe S.M., Lee S., Niculescu-Duvaz D., Good V.M., Project C.G., Jones C.M., Marshall C.J., Springer C.J. (2004). Mechanism of activation of the RAF-ERK signaling pathway by oncogenic mutations of B-RAF. Cell.

[165] Paton E.L., Turner J.A., Schlaepfer I.R. (2020). Overcoming Resistance to Therapies Targeting the MAPK Pathway in BRAF-Mutated Tumours. J. Oncol..

[166] Kim S.J., Lee K.E., Myong J.P., Park J.H., Jeon Y.K., Min H.S., Park S.Y., Jung K.C., Koo D.H., Youn Y.K. (2012). BRAFV600Emutation is associated with tumor aggressiveness in papillary thyroid cancer. World J. Surg..

[167] Choi E.K., Chong A., Ha J.-M., Jung C.K., O J.H., Kim S.H. (2017). Clinicopathological characteristics including BRAF V600E mutation status and PET/CT findings in papillary thyroid carcinoma. Clin. Endocrinol..

[168] Ishizaka Y., Itoh F., Tahira T., Ikeda I., Sugimura T., Tucker J., Fertitta A., Carrano A.V., Nagao M. (1989). Human ret proto-oncogene mapped to chromosome 10q11.2. Oncogene.

[169] Takaya K., Yoshimasa T., Arai H., Tamura N., Miyamoto Y., Itoh H., Nakao K. (1996). Expression of the RET proto-oncogene in normal human tissues, pheochromocytomas, and other tumors of neural crest origin. J. Mol. Med..

[170] Grieco M., Santoro M., Berlingieri M.T.M., Melillo R.M.R., Donghi R., Bongarzone I., Pierotti M.A., Della Porta G., Fusco A., Vecchio G. (1990). PTC is a novel rearranged form of the ret proto-oncogene and is frequently detected in vivo in human thyroid papillary carcinomas. Cell.

[171] Staubitz J.I., Schad A., Springer E., Rajalingam K., Lang H., Roth W., Hartmann N., Musholt T.J. (2019). Novel rearrangements involving the RET gene in papillary thyroid carcinoma. Cancer Genet..

[172] Rabes H.M., Demidchik E.P., Sidorow J.D., Implications C., Lengfelder E., Beimfohr C., Hoelzel D. (2000). Pattern of Radiation-induced RET and NTRK1 Rearrangements in 191 Post-Chernobyl Papillary Thyroid Carcinomas: Biological, Phenotypic, and Clinical Implications. Clin. Res. Cancer.

[173] Zhu Z., Ciampi R., Nikiforova M.N., Gandhi M., Nikiforov Y.E. (2006). Prevalence of RET/PTC Rearrangements in Thyroid Papillary Carcinomas: Effects of the Detection Methods and Genetic Heterogeneity. J. Clin. Endocrinol. Metab..

[174] Khan M.S., Qadri Q., Makhdoomi M.J., Wani M.A., Malik A.A., Niyaz M., Masoodi S.R., Andrabi K.I., Ahmad R., Mudassar S. (2020). RET/PTC Gene Rearrangements in Thyroid Carcinogenesis: Assessment and Clinico-Pathological Correlations. Pathol. Oncol. Res..

[175] Bounacer A., Wicker R., Caillou B., Cailleux A.F., Sarasin A., Schlumberger M., Suárez H.G. (1997). High prevalence of activating ret proto-oncogene rearrangements, in thyroid tumors from patients who had received external radiation. Oncogene.

[176] Adeniran A.J., Zhu Z., Gandhi M., Steward D.L., Fidler J.P., Giordano T.J., Biddinger P.W., Nikiforov Y.E. (2006). Correlation Between Genetic Alterations and Microscopic Features, Clinical Manifestations, and Prognostic Characteristics of Thyroid Papillary Carcinomas. Am. J. Surg. Pathol..

[177] Koo J.S., Hong S., Park C.S. (2009). Diffuse sclerosing variant is a major subtype of papillary thyroid carcinoma in the young. Thyroid.

[178] Malandrino P., Russo M., Regalbuto C., Pellegriti G., Moleti M., Caff A., Squatrito S., Vigneri R. (2016). Outcome of the Diffuse Sclerosing Variant of Papillary Thyroid Cancer: A Meta-Analysis. Thyroid.

[179] Elisei R., Romei C., Vorontsova T., Cosci B., Veremeychik V., Kuchinskaya E., Basolo F., Demidchik E.P., Miccoli P., Pinchera A. (2001). RET/PTC rearrangements in thyroid nodules: Studies in irradiated and not irradiated, malignant and benign thyroid lesions in children and adults. J. Clin. Endocrinol. Metab..

[180] Prescott J.D., Zeiger M.A. (2015). The RET oncogene in papillary thyroid carcinoma. Cancer.

[181] Kroll T.G., Sarraf P., Pecciarini L., Chen C.J., Mueller E., Spiegelman B.M., Fletcher J.A. (2000). PAX8-PPARγ1 fusion in oncogene human thyroid carcinoma. Science.

[182] Ballester L.Y., Sarabia S.F., Sayeed H., Patel N., Baalwa J., Athanassaki I., Hernandez J.A., Fang E., Quintanilla N.M., Roy A. (2016). Integrating Molecular Testing in the Diagnosis and Management of Children with Thyroid Lesions. Pediatr. Dev. Pathol..

[183] Nikiforova M.N., Lynch R.A., Biddinger P.W., Alexander E.K., Dorn G.W., Tallini G., Kroll T.G., Nikiforov Y.E. (2003). RAS point mutations and PAX8-PPARγ rearrangement in thyroid tumors: Evidence for distinct molecular pathways in thyroid follicular carcinoma. J. Clin. Endocrinol. Metab..

[184] Marques A.R., Espadinha C., Catarino A.L., Moniz S., Pereira T., Sobrinho L.G., Leite V. (2002). Expression of PAX8-PPARγ1 Rearrangements in Both Follicular Thyroid Carcinomas and Adenomas. J. Clin. Endocrinol. Metab..

[185] Dwight T., Thoppe S.R., Foukakis T., Lui W.O., Wallin G., Höög A., Frisk T., Larsson C., Zedenius J. (2003). Involvement of the PAX8/peroxisome proliferator-activated receptor γ rearrangement in follicular thyroid tumors. J. Clin. Endocrinol. Metab..

[186] Marshall C.J., Vousden K., Ozanne B. (1985). The involvement of activated ras genes in determining the transformed phenotype. Proc. R. Soc. Lond. Biol. Sci..

[187] Nikiforov Y.E., Nikiforova M.N., Gnepp D.R., Fagin J.A. (1996). Prevalence of mutations of ras and p53 in benign and malignant thyroid tumors from children exposed to radiation after the Chernobyl nuclear accident—PubMed. Oncogene.

[188] Jang E.K., Song D.E., Sim S.Y., Kwon H., Choi Y.M., Jeon M.J., Han J.M., Kim W.G., Kim T.Y., Shong Y.K. (2014). NRAS codon 61 mutation is associated with distant metastasis in patients with follicular thyroid carcinoma. Thyroid.

[189] Cleal K., Norris K., Baird D. (2018). Telomere length dynamics and the evolution of cancer genome architecture. Int. J. Mol. Sci..

[190] Maciejowski J., De Lange T. (2017). Telomeres in cancer: Tumour suppression and genome instability. Nat. Rev. Mol. Cell Biol..

[191] De Lange T. (2005). Shelterin: The protein complex that shapes and safeguards human telomeres. Genes Dev..

[192] di Fagagna F.D. (2008). Living on a break: Cellular senescence as a DNA-damage response. Nat. Rev. Cancer.

[193] De Lange T. (2009). How telomeres solve the end-protection problem. Science.

[194] Sobinoff A.P., Pickett H.A. (2017). Alternative Lengthening of Telomeres: DNA Repair Pathways Converge. Trends Genet..

[195] De Vitis M., Berardinelli F., Sgura A. (2018). Telomere length maintenance in cancer: At the crossroad between telomerase and alternative lengthening of telomeres (ALT). Int. J. Mol. Sci..

[196] Venturini L., Daidone M.G., Motta R., Collini P., Spreafico F., Terenziani M., Piva L., Radice P., Perotti D., Zaffaroni N. (2011). Telomere maintenance in wilms tumors: First evidence for the presence of alternative lengthening of telomeres mechanism. Genes Chromosom. Cancer.

[197] Hakin-Smith V., Jellinek D.A., Levy D., Carroll T., Teo M., Timperley W.R., McKay M.J., Reddel R.R., Royds J.A. (2003). Alternative lengthening of telomeres and survival in patients with glioblastoma multiforme. Lancet.

[198] Omori Y., Nakayama F., Li D., Kanemitsu K., Semba S., Ito A., Yokozaki H. (2009). Alternative lengthening of telomeres frequently occurs in mismatch repair system-deficient gastric carcinoma. Cancer Sci..

[199] Ulaner G.A., Hoffman A.R., Otero J., Huang H.-Y., Zhao Z., Mazumdar M., Gorlick R., Meyers P., Healey J.H., Ladanyi M. (2004). Divergent patterns of telomere maintenance mechanisms among human sarcomas: Sharply contrasting prevalence of the alternative lengthening of telomeres mechanism in Ewing’s sarcomas and osteosarcomas. Genes Chromosom. Cancer.

[200] Else T., Giordano T.J., Hammer G.D. (2008). Evaluation of telomere length maintenance mechanisms in adrenocortical carcinoma. J. Clin. Endocrinol. Metab..

[201] Villa R., Daidone M.G., Motta R., Venturini L., De Marco C., Vannelli A., Kusamura S., Baratti D., Deraco M., Costa A. (2008). Multiple mechanisms of telomere maintenance exist and differentially affect clinical outcome in diffuse malignant peritoneal mesothelioma. Clin. Cancer Res..

[202] Xu B., Peng M., Song Q. (2014). The co-expression of telomerase and ALT pathway in human breast cancer tissues. Tumor Biol..

[203] Xue Y., Li L., Zhang D., Wu K., Chen Y., Zeng J., Wang X., He D. (2011). Twisted Epithelial-to-Mesenchymal Transition Promotes Progression of Surviving Bladder Cancer T24 Cells with hTERT-Dysfunction. PLoS ONE.

[204] Bojovic B., Booth R.E., Jin Y., Zhou X., Crowe D.L. (2015). Alternative lengthening of telomeres in cancer stem cells in vivo. Oncogene.

[205] Barthel F.P., Wei W., Tang M., Martinez-Ledesma E., Hu X., Amin S.B., Akdemir K.C., Seth S., Song X., Wang Q. (2017). Systematic analysis of telomere length and somatic alterations in 31 cancer types. Nat. Genet..

[206] Castelo-Branco P., Leão R., Lipman T., Campbell B., Lee D., Price A., Zhang C., Heidari A., Stephens D., Boerno S. (2016). A cancer specific hypermethylation signature of the TERT promoter predicts biochemical relapse in prostate cancer: A retrospective cohort study. Oncotarget.

[207] Donati B., Ciarrocchi A. (2019). Telomerase and telomeres biology in thyroid cancer. Int. J. Mol. Sci..

[208] Liu C., Liu Z., Chen T., Zeng W., Guo Y., Huang T. (2016). TERT promoter Mutation and Its Association with Clinicopathological Features and Prognosis of Papillary Thyroid Cancer: A Meta-analysis. Sci. Rep..

[209] Geng J., Liu Y., Guo Y., Wang H., Tai J., Jin Y., Zhang J., Yu Y., Wang S., Song Y. (2019). Correlation between TERT C228T and clinic-pathological features in pediatric papillary thyroid carcinoma. Sci. China Life Sci..

[210] Chiba K., Lorbeer F.K., Shain A.H., McSwiggen D.T., Schruf E., Oh A., Ryu J., Darzacq X., Bastian B.C., Hockemeyer D. (2017). Mutations in the promoter of the telomerase gene TERT contribute to tumorigenesis by a two-step mechanism. Science.

[211] Mai S., Garini Y. (2006). The significance of telomeric aggregates in the interphase nuclei of tumor cells. J. Cell. Biochem..

[212] Gadji M., Pozzo A.R. (2019). From cellular morphology to molecular and epigenetic anomalies of myelodysplastic syndromes. Genes Chromosom. Cancer.

[213] Caria P., Dettori T., Frau D.V., Lichtenzstejn D., Pani F., Vanni R., Mai S. (2019). Characterizing the three-dimensional organization of telomeres in papillary thyroid carcinoma cells. J. Cell. Physiol..

[214] Mai S. (2019). The three-dimensional cancer nucleus. Genes Chromosom. Cancer.

[215] Belmont A.S., Zhai Y., Thilenius A. (1993). Lamin B distribution and association with peripheral chromatin revealed by optical sectioning and electron microscopy tomography. J. Cell Biol..

[216] Belmont A.S., Bignone F., Ts’O P.O.P. (1986). The relative intranuclear positions of barr bodies in XXX non-transformed human fibroblasts. Exp. Cell Res..

[217] Fritz A.J., Sehgal N., Pliss A., Xu J., Berezney R. (2019). Chromosome territories and the global regulation of the genome. Genes Chromosom. Cancer.

[218] Nikiforova M.N., Stringer J.R., Blough R., Medvedovic M., Fagin J.A., Nikiforov Y.E. (2000). Proximity of chromosomal loci that participate in radiation-induced rearrangements in human cells. Science.

[219] Gandhi M., Medvedovic M., Stringer J.R., Nikiforov Y.E. (2006). Interphase chromosome folding determines spatial proximity of genes participating in carcinogenic RET/PTC rearrangements. Oncogene.

[220] Elder A.D., Domin A., Kaminski Schierle G.S., Lindon C., Pines J., Esposito A., Kaminski C.F. (2009). A quantitative protocol for dynamic measurements of protein interactions by Förster resonance energy transfer-sensitized fluorescence emission. J. Royal Soc. Interface.

[221] Zink D., Fischer A.H., Nickerson J.A. (2004). Nuclear structure in cancer cells. Nat. Rev. Cancer.

[222] Wegel E., Göhler A., Lagerholm B.C., Wainman A., Uphoff S., Kaufmann R., Dobbie I.M. (2016). Imaging cellular structures in super-resolution with SIM, STED and Localisation Microscopy: A practical comparison. Sci. Rep..

[223] Heintzmann R., Cremer C.G., Bigio I.J., Schneckenburger H., Slavik J., Svanberg K., Viallet P.M. (1999). Laterally modulated excitation microscopy: Improvement of resolution by using a diffraction grating. Optical Biopsies and Microscopic Techniques III.

[224] Gustafsson M.G.L., Shao L., Carlton P.M., Wang C.J.R., Golubovskaya I.N., Cande W.Z., Agard D.A., Sedat J.W. (2008). Three-dimensional resolution doubling in wide-field fluorescence microscopy by structured illumination. Biophys. J..

[225] Gustafsson M.G.L. (2000). Surpassing the lateral resolution limit by a factor of two using structured illumination microscopy. J. Microsc..

[226] Righolt C.H., Guffei A., Knecht H., Young I.T., Stallinga S., Van Vliet L.J., Mai S. (2014). Differences in nuclear DNA organization between lymphocytes, hodgkin and reed-sternberg cells revealed by structured illumination microscopy. J. Cell. Biochem..

[227] Righolt C.H., Knecht H., Mai S. (2016). DNA Superresolution Structure of Reed-Sternberg Cells Differs between Long-Lasting Remission versus Relapsing Hodgkin’s Lymphoma Patients. J. Cell. Biochem..

[228] Sathitruangsak C., Righolt C.H., Klewes L., Tammur P., Ilus T., Tamm A., Punab M., Olujohungbe A., Mai S. (2015). Quantitative superresolution microscopy reveals differences in nuclear dna organization of multiple myeloma and monoclonal gammopathy of undetermined significance. J. Cell. Biochem..

[229] Rangel-Pozzo A., Booth S., Yu P.L.I., Singh M., Selivanova G., Mai S. (2020). p53 CRISPR Deletion Affects DNA Structure and Nuclear Architecture. J. Clin. Med..

[230] Rangel-Pozzo A., Kuzyk A., Gartner J., Mai S. (2019). MYCN overexpression is linked to significant differences in nuclear DNA organization in neuroblastoma. SPG BioMed.

[231] Ajaezi G.C., Eisele M., Contu F., Lal S., Rangel-Pozzo A., Mai S., Gough K.M. (2018). Near-field infrared nanospectroscopy and super-resolution fluorescence microscopy enable complementary nanoscale analyses of lymphocyte nuclei. Analyst.

